# Mineral nitrogen sources differently affect root glutamine synthetase isoforms and amino acid balance among organs in maize

**DOI:** 10.1186/s12870-015-0482-9

**Published:** 2015-04-03

**Authors:** Bhakti Prinsi, Luca Espen

**Affiliations:** Dipartimento di Scienze Agrarie e Ambientali - Produzione, Territorio, Agroenergia (DISAA), Università degli Studi di Milano, Via Celoria, 2, 20133 Milano, Italy

**Keywords:** Amino acids, Ammonium, Glutamine synthetase, Maize, Nitrate, Roots

## Abstract

**Background:**

Glutamine synthetase (GS) catalyzes the first step of nitrogen assimilation in plant cell. The main GS are classified as cytosolic GS1 and plastidial GS2, of which the functionality is variable according to the nitrogen sources, organs and developmental stages. In maize (*Zea mays* L.) one gene for GS2 and five genes for GS1 subunits are known, but their roles in root metabolism are not yet well defined. In this work, proteomic and biochemical approaches have been used to study root GS enzymes and nitrogen assimilation in maize plants re-supplied with nitrate, ammonium or both.

**Results:**

The plant metabolic status highlighted the relevance of root system in maize nitrogen assimilation during both nitrate and ammonium nutrition. The analysis of root proteomes allowed a study to be made of the accumulation and phosphorylation of six GS proteins. Three forms of GS2 were identified, among which only the phosphorylated one showed an accumulation trend consistent with plastidial GS activity. Nitrogen availabilities enabled increments in root total GS synthetase activity, associated with different GS1 isoforms according to the nitrogen sources. Nitrate nutrition induced the specific accumulation of GS1-5 while ammonium led to up-accumulation of both GS1-1 and GS1-5, highlighting co-participation. Moreover, the changes in thermal sensitivity of root GS transferase activity suggested differential rearrangements of the native enzyme. The amino acid accumulation and composition in roots, xylem sap and leaves deeply changed in response to mineral sources. Glutamine showed the prevalent changes in all nitrogen nutritions. Besides, the ammonium nutrition was associated with an accumulation of asparagine and reducing sugars and a drop in glutamic acid level, significantly alleviated by the co-provision with nitrate.

**Conclusion:**

This work provides new information about the multifaceted regulation of the GS enzyme in maize roots, indicating the involvement of specific isoenzymes/isoforms, post-translational events and biochemical factors. For the first time, the proteomic approach allowed to discriminate the individual contribution of the GS1 isoforms, highlighting the participation of GS1-5 in nitrate metabolism. Moreover, the results give new insights about the influence of amino acid metabolism in plant C/N balance.

**Electronic supplementary material:**

The online version of this article (doi:10.1186/s12870-015-0482-9) contains supplementary material, which is available to authorized users.

## Background

Nitrogen (N) represents one of the main minerals required throughout plant development. In agronomic terms, this results in a worldwide ever-increasing use of fertilizers and its consequent environmental and socioeconomic costs [1]. This N requirement is emphasized with regard to cereal crops [2], for which maize (*Zea mays* L.) is a model species because of its economic importance and high metabolic capacity [3]. In agricultural soils the main mineral N sources are nitrate (NO_3_^−^) and ammonium (NH_4_^+^). In order to balance their N nutritional requirements with environmental availability, plants have to modulate the individual steps of N metabolism such as up-take, reduction of NO_3_^−^ to NH_4_^+^, NH_4_^+^ assimilation and N recycling. The contribution of root and leaf systems depends on species, developmental stage and environmental conditions [4,5], and it is also deeply influenced by C metabolism [6].

All the NH_4_^+^ in the cell, derived from soil, from NO_3_^−^ reduction or from other metabolic processes, is channelled through the glutamine synthetase (GS, EC6.3.1.2) reaction. The GS catalyzes the fixation of NH_4_^+^ on glutamic acid (Glu) to form glutamine (Gln), and in the assimilation process it is generally coupled with plastidial glutamate synthase (GOGAT, EC1.4.1.13/14) that incorporates C skeletons. Gln and Glu can be recruited as amino group donors as well as main N transport molecules [7]. Several evidence indicate that GS activity is deeply influenced by metabolic and environmental factors mainly linked to the balance between C and N metabolism [8]. For instance, Glu level seems to be fundamental in sensing plant nutritional status and in joining C and N metabolisms [9]. Moreover, the inter-conversion with other amino acids greatly influences N plant economy, especially regarding asparagine (Asn) and alanine (Ala) [10].

Plant responses are deeply affected by the proportion of mineral N sources [11]. While NH_4_^+^ as sole nutrient can induce toxicity symptoms, its co-provision with NO_3_^−^ generally promotes a synergistic effect leading to growth enhancement [12]. It is noteworthy that NH_4_^+^ tolerance was related to high root N metabolism sustained by high GS activities [13], which in maize appear to be associated with the capacity to cope with the C skeleton demands [14].

The main GS are decameric enzymes [15] classified on the basis of subcellular localization in cytosolic GS1 and plastidial GS2. In plants, multigenic families encode several GS1 isoforms while the plastidial GS2 derives from one or few nuclear genes. In general, GS2 is associated with the leaf NH_4_^+^ (re)assimilation while GS1 is associated with plant N recycling. But the relative activity of GS1 and GS2 is variable according to the species, organs, N sources, developmental stages and environmental conditions, suggesting a multifaceted participation of isozymes [16]. Moreover, recent studies conducted both in dicotyledonous [17] and in monocotyledonous crops [18,19] showed non-overlapping functions for the GS1 isoforms. Besides, distinct post-translational modifications were described for both isoenzymes [20,21].

In maize, one gene for GS2 [SwissProt:P25462] and 5 genes codifying for different GS1 subunits were identified, named from GS1-1 to GS1-5 according to the reviewed UniProtKB/Swiss-Prot database [22] [Swiss-Prot:P38559; Swiss-Prot:P38560; Swiss-Prot:P38561; Swiss-Prot:P38562; Swiss-Prot:P38563]. GS1 and GS2 are differentially regulated in roots and leaves in response to growing conditions. The cytosolic isoforms also have different kinetic properties, stabilities and tissue localizations [23-25]. By means of Quantitative Trait Loci analyses and characterization of maize mutants, Hirel and co-workers indicated the key roles of GS1-3 and GS1-4 both in grain yield and germination [19,26]. GS1-3 and GS1-4 represent the major leaf isoforms [19] and in maize mutants the deficiency of these enzymes affects leaf gene transcripts, proteins and metabolite accumulations [27]. Moreover, the transcript localizations confirmed the involvement of GS1-1 in root metabolism and suggested that GS1-2 acts in N phloem translocation [19]. It is worth noting that in mutants deficient for GS1-3 and GS1-4 the dry weight and total N content in the shoot vegetative parts were unaltered, providing evidence of how such parameters are prevalently determined by root metabolism [19]. This observation, together with the finding that the N stored before silking supplies up to 70% of the grain N content [28], draws attention to the need to study the root system during the early phases of maize development. After a first localization of GS1-1 and GS1-5 in tip and/or cortex tissues [25], root GS arrangement had scarcely been investigated, probably due to technical limitations. N availability is associated with the accumulation of a specific root isoform (GSr in [24]) that is theoretically assigned to GS1-1, but had not been precisely characterized. Similarly, the responses to different N sources were not fully elucidated and information about GS1-5 was still lacking.

By the means of Two-Dimensional Western Blotting (2D-WB) and Liquid Chromatography-nanoElectroSpray Ionization-Tandem Mass Spectrometry (LC-nESI-MS/MS) techniques, this work profiles the GS patterns in maize roots in response to NO_3_^−^, NH_4_^+^ or both, during vegetative growth, describing for the first time the differential modulations of cytosolic and plastidial forms and the active involvement of GS1-5. Moreover, the determination of amino acid composition in roots, xylem sap and leaves provides new information about the roles of Gln, Glu, Asn, and Ala metabolisms in plant C/N balance.

## Results and discussion

### Effects of the nutritional treatments on leaf and root metabolic status

The aim of this work was to investigate the responses of the different root GS isoforms in maize plants exposed to different inorganic N sources, during early vegetative growth. To better appreciate the effects at metabolic level, the changes in plant amino acid balance were also evaluated. Plantlets of the T250 inbred line were grown in a hydroponic system in the absence of N for 10 days to reach a developmental stage corresponding to the third-leaf expansion (Additional file 1: Figure A1 and Table A1). Since in field conditions maize N fertilization consists of a single application at sowing [29], the third-leaf stage corresponds to a vegetative phase in which plants are exposed to a high level of inorganic N and that is indicated as one of the more susceptible to NH_4_^+^ toxicity [14]. Moreover, it is important to note that the optimal dose of NO_3_^−^ fertilization also depends on maize varieties [30]. In order to better appreciate the short-term responses and compare our proteomic results with previous works, the plants were exposed to a total N availability of 10 mM. In details, plants were exposed for 30 h to four nutritional treatments: N absence (c), 10 mM NO_3_^−^ (n), 10 mM NH_4_^+^ (a), 5 mM NO_3_^−^ + 5 mM NH_4_^+^ (na).

**Figure 1:**
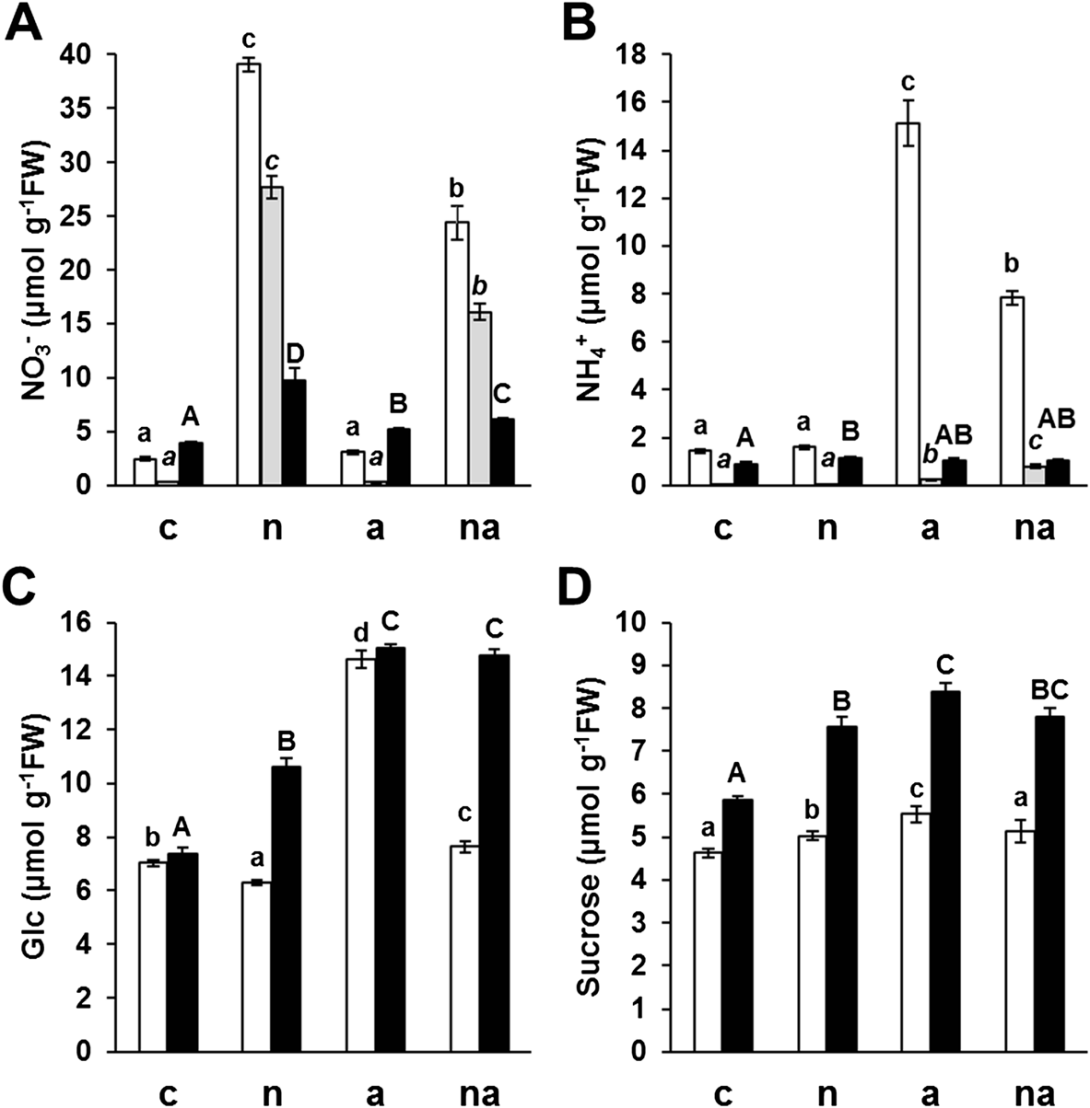
**Nitrogen and carbon metabolites in maize in response to different inorganic N sources.** Concentration of NO_3_^−^ **(A)**, NH_4_^+^ **(B)**, reducing sugars **(C)** and sucrose **(D)** in roots (white bars), xylem sap (grey bars) and leaves (black bars) in maize plants grown for 10 days without N sources and then exposed for the last 30 h to absence of N (c), to 10 mM NO_3_^−^ (n), to 10 mM NH_4_^+^ (a) or to 5 mM NO_3_^−^ + 5 mM NH_4_^+^ (na). Graphs show average values ± SE (n = 6). The upper letters indicate differences among the four treatments within each organ according to Student’s t-test (p < 0.05).

**Figure 2:**
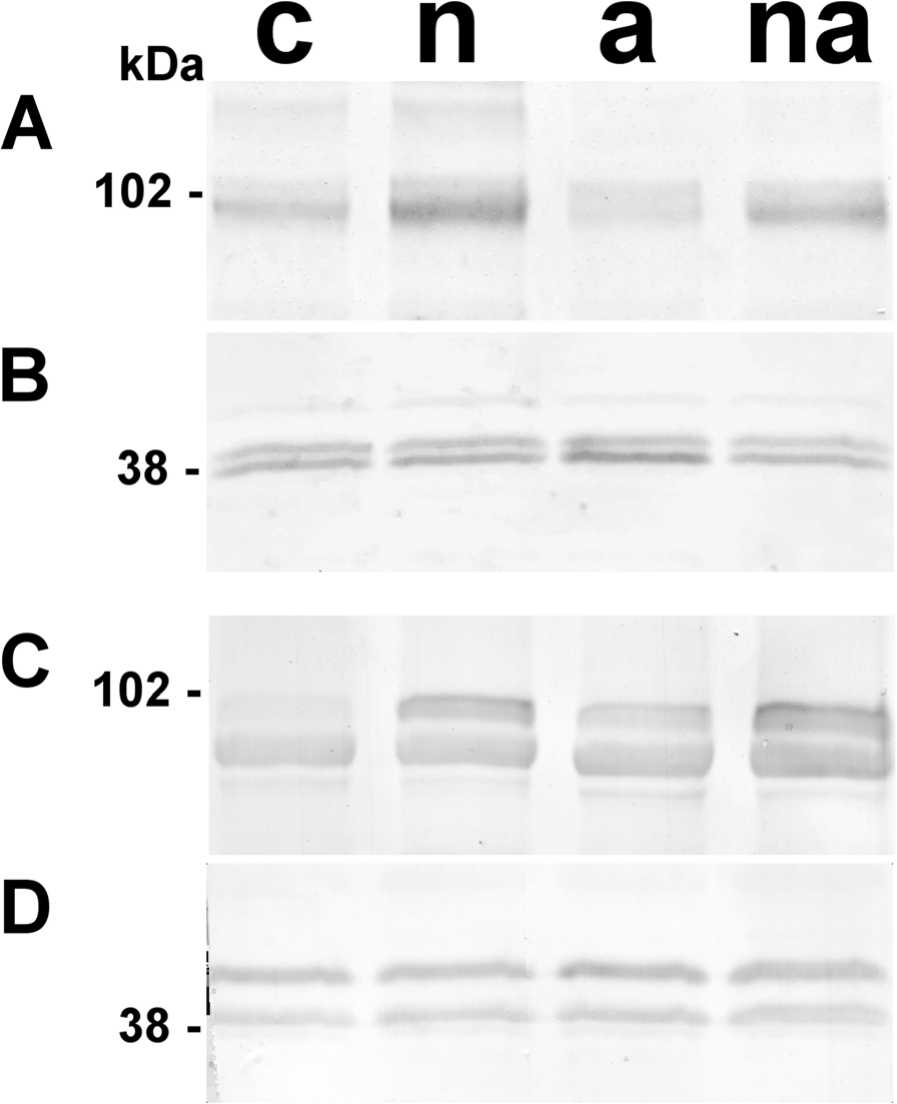
**Accumulation of N assimilatory enzymes in maize in response to inorganic N sources.** 1D-WB performed against NR **(A, C)** and GS **(B, D)** in roots **(A, B)** and leaves **(C, D)** of maize plants grown without N sources for 10 days and then exposed for the last 30 h to absence of N (c), to 10 mM NO_3_^−^ (n), to 10 mM NH_4_^+^ (a) or to 5 mM NO_3_^−^ + 5 mM NH_4_^+^ (na). The analyses were conducted twice on two independent biological samples (n = 4) for each condition. **B** and **D** profiles were visualized by a global antibody for both GS1 and GS2 that recognized three different bands. The molecular masses of electrophoretic standards are reported on the left (kDa).

The concentration of NO_3_^−^ and NH_4_^+^ in roots, xylem saps and leaves were measured (Figure 1A and B), together with the content of the reducing sugars and sucrose in root and leaf systems (Figure 1C and D). Moreover, the accumulation of the main N assimilative enzymes such as Nitrate Reductase (NR, EC 1.6.6.1; [31]) and GS in roots and leaves was estimated by One-Dimensional Western Blotting (1D-WB) (Figure 2). The analysis conducted against NR in roots detected the expected single band at 99 kDa (Figure 2A) while in leaf profiles two bands were visible (Figure 2C). The lower band at about 94 kDa, corresponding to pyruvate phosphate dikinase (EC2.7.9.1) that is the most abundant enzyme in maize leaves (Additional file 1: Figure A2), was considered as an unspecific signal. On the base of molecular masses, the three bands in GS profiles (Figure 2B and D) were assigned to GS2 (44 kDa), to GS1 (40 kDa) and to the root isoform GS1r (39 kDa), as described by Sakakibara and co-workers [23]. According to previous works [19,23,32], higher levels of GS1 were detected in root profiles (Figure 2B) while GS2 was predominant in leaves (Figure 2D).

It appeared evident that the exposure of the plants to NO_3_^−^ induced strong accumulation of the anion in all organs and especially in roots, reaching the value of 39.08 ± 0.62 μmol g^−1^FW in (n) plants (Figure 1A). This trend was similar to that observed in T250 by previous time-course experiments [32]. The increment of NO_3_^−^ in xylem sap attested the concomitant induction of anion translocation. In particular, in (n) and (na) xylem saps the values of 27.70 ± 1.02 and 16.10 ± 0.71 μmol g^−1^FW, respectively (Figure 1A), quite a lot higher than the 10.5 mM value observed in maize crops [33], suggested an extensive plant response to restoring tissue N levels. The N management in plants exposed to NH_4_^+^ was different. Both (a) and (na) plants did not show any toxicity symptoms and the NH_4_^+^ root accumulation and translocation incremented proportionally to the medium concentrations (Figure 1B). On the contrary, the NH_4_^+^ levels in leaf systems were scarcely affected especially in comparison with roots in which NH_4_^+^ reached the highest value of 15.13 ± 0.93 μmol g^−1^FW in (a) condition (Figure 1B). In several plant species, a similar limitation of NH_4_^+^ translocation to the shoot, considered more sensitive to NH_4_^+^ than roots [12], was associated to a high rate of metabolisation by GS in roots, which was proposed as one of the main traits of tolerance to high NH_4_^+^ inputs [13,34]. Finally, no synergistic effect on the ions accumulation emerged (Figure 1).

The availability of N led to an increase of both reducing sugars and sucrose levels in leaves, especially in response to NH_4_^+^ exposure (Figure 1C and D). However, only in the roots of (a) plants a peculiar doubling of reducing sugars (14.63 ± 0.32 μmol g^-1^FW) was observed (Figure 1C). Considering that the sucrose slightly increased, it is possible that this increment originated from the delivery of photo-assimilates from the shoot. Interestingly, this trait was almost completely alleviated by the co-provision with NO_3_^−^ (Figure 1C). In a comparative study among maize genotypes, the physiological traits of NH_4_^+^ tolerance seemed to rely on the plant’s capability to shift the partitioning of carbohydrates towards the root system in order to sustain NH_4_^+^ assimilation [14]. Hence, the results confirmed that the T250 line shows some physiological traits typical of tolerant maize cultivars, especially those deriving from a high capability for NH_4_^+^ assimilation by the root system. At the same time, the reduction of reducing sugars in (n) roots (6.29 ± 0.09 μmol g^−1^FW) indicated that the roots were also involved in NO_3_^−^ assimilation, as proposed by Prinsi and co-workers [32].

The 1D-WB supported the induction of the assimilatory pathways (Figure 2). The NR accumulated proportionally to the NO_3_^−^ concentration in the external medium both in roots and leaves (Figure 2A and C). Similarly, the GS2 levels in roots (Figure 2B) responded to NO_3_^−^ availability. This response was scarcely detected in leaves, where the enzyme level was quite constant (Figure 2D). This behaviour is similar to that observed in young vegetative maize plants grown at high and low N fertilization in the field [35]. Interestingly, in comparison to (c) plants, the exposure to NH_4_^+^ led to a reduction of NR level in roots and to a slight increment in leaves. Together with the increment of the NO_3_^−^ accumulated in the shoot (5.16 ± 0.12 μmol g^−1^FW, Figure 1A), these results suggest that the NH_4_^+^ availability induced a shift in plant N economy, promoting the usage of NO_3_^−^ reserves by the shoot. Finally, the GS1 enzyme accumulated in response to the increasing cell NH_4_^+^ contents, by the specific induction of the GSr isoform (Figure 2B and D).

In summary, the plant metabolic status results confirmed that the four experimental treatments induced the assimilation and translocation of the different N sources, highlighting the fundamental role of the root system in the plant physiological adaptation to both NO_3_^−^ and NH_4_^+^ nutrition.

### Characterization of the GS isoforms in maize roots

**Figure 3:**
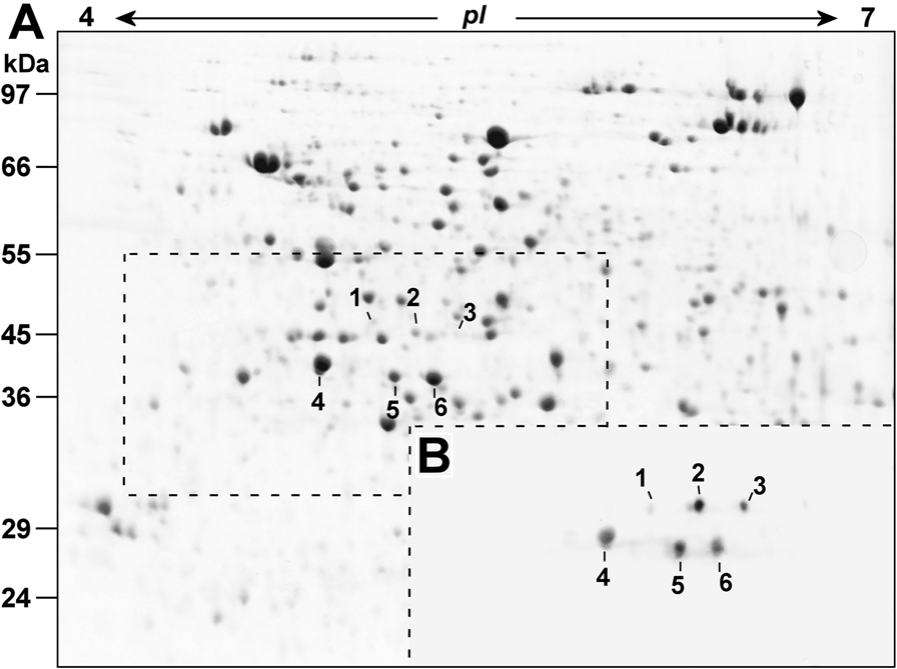
**Localization of the GS isoforms in 2D profile of maize roots by Western Blotting.** Representative 2D-Electrophoretic map of soluble protein fraction from roots of maize plants. Proteins were analyzed by isoelectric focusing at pH 4–7 and 10% SDS-PAGE and visualized by cCBB staining **(A)**. For each nutritional treatments, the gel portion comprised in the broken-line was analyzed by WB against all GS (**B**, GS1 + GS2 global antibody). The analyses were conducted twice on two independent biological samples (n = 4) showing the same profile among all the experimental conditions. The visualized spots are numbered (1–6) and traced on the 2D-Electrophoretic map. The LC-nESI-MS/MS characterizations of the six spots are reported in Table 1. The molecular masses of electrophoretic standards are reported on the left (kDa).

In order to distinguish at protein level the different GS isoforms accumulated in the maize root system, the WB with the GS global antibody (GS1 + GS2) was applied on the 2D-electrophoretic map of root soluble proteins. A preliminary investigation conducted in a wide pH range of 3–10 on 10% acrylamide Sodium Dodecyl Sulphate-PolyAcrylamide Gel Electrophoresis (SDS-PAGE) indicated that all recognizable isoforms were embraced in the acidic portion of the profile and in a molecular range of 35–50 kDa (*i.e.* 2D-WB profiles similar to Figure 3). To improve the analytical resolution, the next protein separations were performed in the narrower 4–7 pH range. This approach allowed a good reproducibility among the four nutritional treatments (Additional file 1: Figure A3), providing an overall proteomic map composed of about 1210 ± 26 spots (Figure 3A). Considering the electrophoretic adjustments, this map compared well with the one proposed by Prinsi and co-workers [32]. The 2D-WB showed a unique pattern (Figure 3B), recurrent in all experimental conditions, consisting of six stains that were numbered from 1 to 6 and assigned to six spots in the 2D-electrophoretic maps (Figure 3A). These 24 spots (*i.e.* six per treatment) were separately analysed by LC-nESI-MS/MS. Each spot was assigned to a specific GS isoform according to the discriminating peptides sequenced. Since for every reference spot (Figure 3) the assignment was independently confirmed among the four 2D profiles, the data were then pooled to get the overall spot characterization (Table 1, Additional file 1: Table A2-A7). The spots 1, 2 and 3 were identified as different GS2 forms from the same gene, named GS2a, GS2b and GS2c (Table 1). This observation was in agreement with the fact that two distinct GS2 forms were highlighted in the maize leaf proteome [19]. Here, as well as in several other plant species, the GS2 protein forms exceed the number of encoding genes ([21,36] and references therein). As a whole, this trait supports the presence of post-transcriptional/translational modifications (PTMs, [37]). The similar incidence of specific peptides for both the two isoforms revealed that spot 4 derived from an overlapping of GS1-3 and GS1-4, explainable by the almost identical isoelectric point that made the two proteins inseparable by denaturing electrophoresis (Table 1), as previously noted by Martin and co-workers [19]. Conversely, spots 5 and 6 were distinctly identified as GS1-5 and GS1-1 isoforms, respectively (Table 1). This proteomic investigation revealed a pattern of GS accumulation very consistent with the expression profile proposed by Li and co-workers [25]. These authors, by means of a comprehensive analysis of the transcript levels of the six GS genes in maize, provided evidence that all isoforms were expressed in the roots, with the prevalence of GS1-3, GS1-4 and GS1-1 mRNAs. At the same time, the fact that GS1-2 was characterized as a low abundance and vascular-specific isoform [19,25] might explain why it was not detectable.

**Table 1:** **Spot assignments to the specific isoform of glutamine synthetase (GS) by LC-nESI-MS/MS**

**N**	**Acr**	**Protein AN**	**pI/MW(t)**	**pI/MW(e)**	**u.p. s/t**	**%Cov.**	**Avg n. p.**
**1**	**GS2a**	Glutamine synthetase chloroplastic P25462	6.42/46.0	5.36/47.6	9/9	8.0 ± 2.4	3.2 ± 0.9
**2**	**GS2b**	Glutamine synthetase chloroplastic P25462	6.42/46.0	5.44/45.5	10/10	16.9 ± 1.3	6.7 ± 0.4
**3**	**GS2c**	Glutamine synthetase chloroplastic P25462	6.42/46.0	5.52/45.2	4/4	7.2 ± 0.6	2.7 ± 0.2
4*	**GS1-3/4**	Glutamine synthetase root isozyme 3 P38561	5.24/39.2	5.22/41.6	9/10	35.9 ± 1.8	8.4 ± 0.4
		Glutamine synthetase root isozyme 4 P38562	5.23/39.0		9/12		
**5**	**GS1-5**	Glutamine synthetase root isozyme 5 P38563	5.52/39.3	5.40/39.4	6/14	44.3 ± 1.3	11.2 ± 0.2
**6**	**GS1-1**	Glutamine synthetase root isozyme 1 P38559	5.60/39.2	5.48/39.1	3/17	40.5 ± 1.8	11.2 ± 0.6

**Figure 4:**
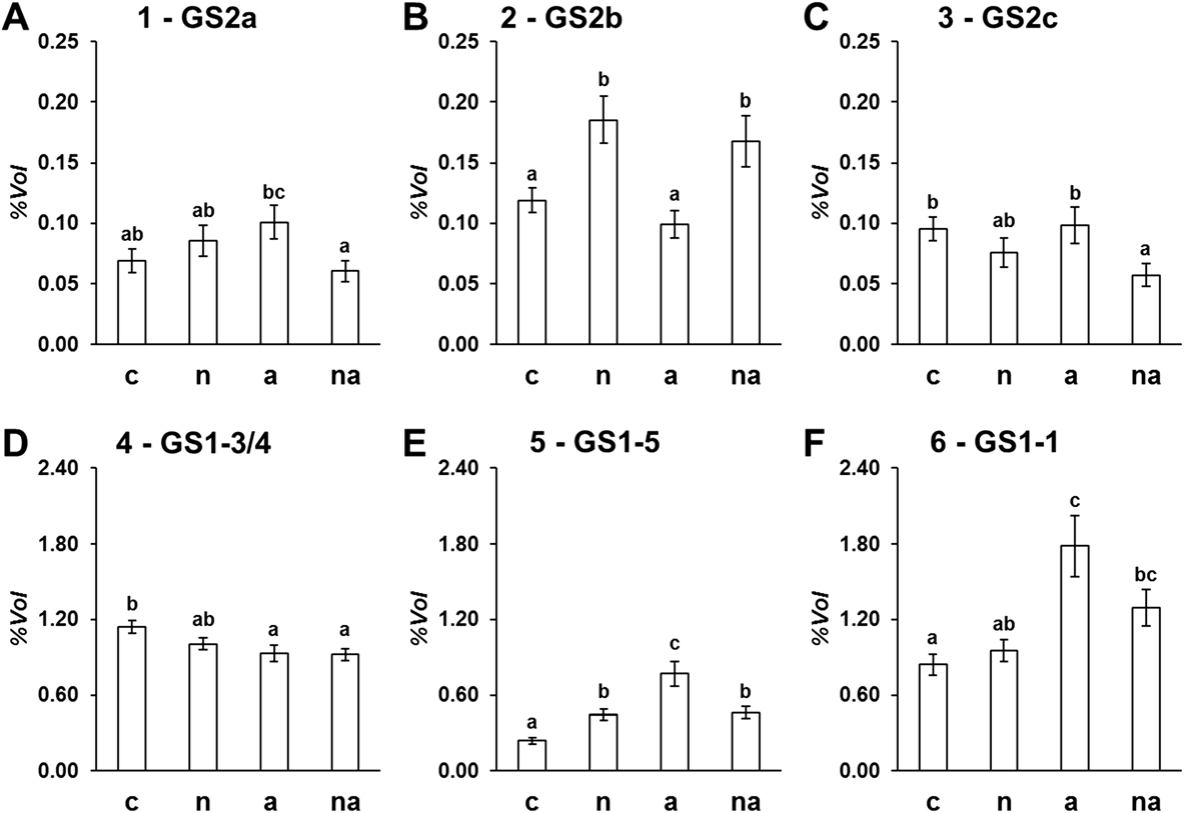
**Accumulation of the GS isoforms in maize roots in response to N sources.** The graphs report the levels *(%Vol*) of the different isoforms evaluated in the root 2D-electrophoretic profiles stained with cCBB (Figure 3) of maize plants grown without N sources for 10 days and then exposed for the last 30 h to absence of N (c), to 10 mM NO_3_^−^ (n), to 10 mM NH_4_^+^ (a) or to 5 mM NO_3_^−^ + 5 mM NH_4_^+^ (na). The graph number and isoform acronyms refer to Figure 3 and Table 1, respectively. **A**. Spot 1 (GS2a). **B**. Spot 2 (GS2b). **C**. Spot 3 (GS2c). **D**. Spot 4 (GS1-3/4). **E**. Spot 5 (GS1-5). **F**. Spot 6 (GS1-1). Graphs show average values ± SE (n = 6). The upper letters indicate differences among the four treatments according to Student’s t-test (p < 0.05).

The different spots indicated in Figure 3 were separately collected from the root 2D profile of each nutritional treatment. Values are the mean ± SE of four independent biological samples (one per treatment) analysed in triplicate (n = 12). 4*: the spot was assigned as a co-migration of the GS1-3 and GS1-4 isoforms. **N**: spot number on Figure 3. **Acr**: acronym reported in Figure 4. **AN**: accession number. **p** ***I*** **/MW** are expressed in kDa and compare the theoretical values (**t**) with the experimental ones (**e**); **u.p.**: number of unique peptides identified, **s**: specific to the isoform, **t**: total. **%Cov**: amino acid coverage. **Avg n. p.:** number of distinct peptides. Detailed information on LC-nESI-MS/MS sequencing is reported in Additional file 1.

**Figure 5:**
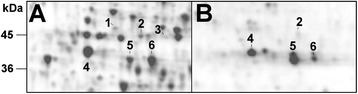
**Analysis of phosphorylation state of root GS isoforms.** The 2D-electrophoretic profiles in 4–7 p*I* range of soluble proteins from root of maize plants grown in the different experimental conditions were stained with sequential fluorescence staining procedures. **A.** Representative magnification of the profile containing the GS isoforms stained with Sypro Ruby®, showing all the proteins. **B.** Representative magnification of the same gel portion after the Pro-Q® Diamond phosphoprotein gel staining that points out only the phosphorylated proteins. The experiment was conducted for each nutritional condition showing a similar profile for all the samples. The spots are numbered (1–6) according to Figure 3 and Table 1. The molecular masses of electrophoretic standards are reported on the left (kDa).

**Figure 6:**
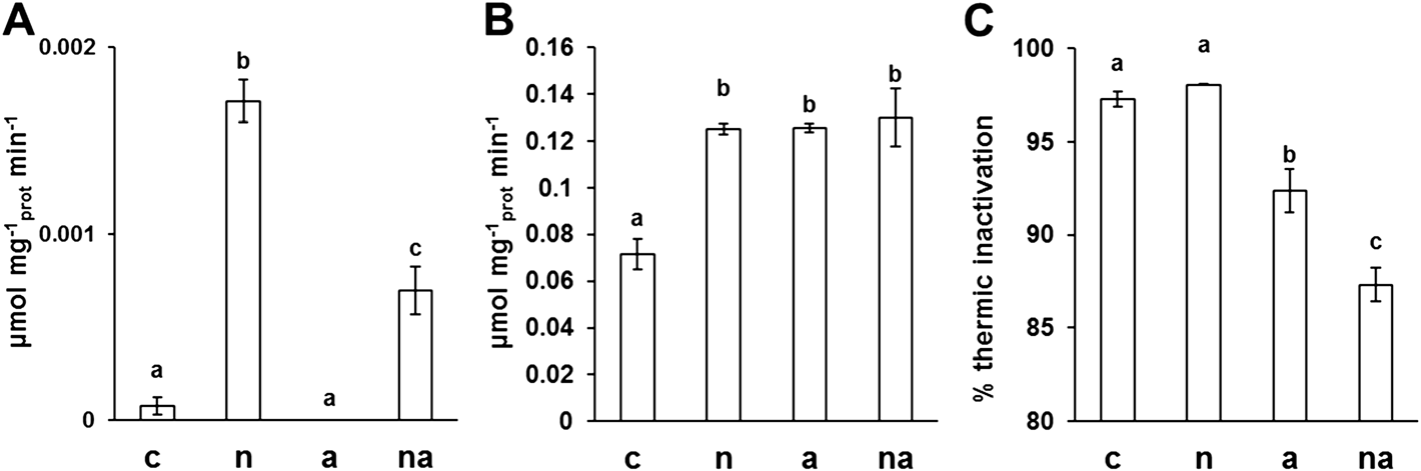
**Effects of the different inorganic N sources on GS activity in maize root.** Maize plants were grown without N sources for 10 days and then exposed for the last 30 h to absence of N (c), to 10 mM NO_3_^−^ (n), to 10 mM NH_4_^+^ (a) or to 5 mM NO_3_^−^ + 5 mM NH_4_^+^ (na). Both the GS synthetase **(A, B)** and the transferase activities **(C)** were evaluated and expressed as specific activity. The synthetase activity was measured in the enriched fraction of plastidial proteins **(A)** and in total protein fraction **(B)** representing the GS2 activity and the total activity, respectively. The transferase activity was measured in total protein fraction prior to and after the exposure of the samples to 45°C for 10 min. The % thermal inactivation **(C)** represents the portion of transferase activity lost after this thermic treatment. Graphs show average values ± SE (n = 3). The upper letters indicate differences among the four treatments according to Student’s t-test (p < 0.05).

Considering the electrophoretic positions of the GS proteins in roots, it was possible to conclude that the middle band observed in the 1D-WB (Figure 2B) included GS1-3 and GS1-4, while the GSr form (Figure 2B), accumulated in response to external N availability, was composed by GS1-1 with GS1-5. This conclusion was previously suggested [24], but to our knowledge, this proteomic approach allowed the first discrimination between the individual responses of GS1-1 and GS1-5. The accumulation levels of the six GS proteins (%*Vol*) in the 2D profiles showed different and specific changes in responses to inorganic N sources (Figure 4). Moreover, the 2D profiles were staining with the Pro-Q® diamond that is specific for phosphoproteins [38], applying this approach to investigate the phosphorylation state of GS isoforms, to our knowledge for the first time. The results showed that four of the six spots were phosphorylated and that the spot phosphorylation state was constant among all conditions (Figure 5). The results, together with the evaluations of root GS activities (Figure 6), contributed to improve the characterization of the six proteins.

### The responses to inorganic N sources of the GS2 proteins in maize roots

GS2 was a very faint protein in the maize roots, with a maximum value of only 0.19 ± 0.02 %*Vol* for GSb in (n) plants (Figure 4). Similarly, the highest GS synthetase activity of the plastidial fraction was detected in (n) plants, where it represented about the 1.4% of the total enzymatic activity (Figure 6A and B). This proportion appeared similar to that measured in rice (*Oryza sativa* L.) roots, in which the contribution of GS2 was less than 4% of the total GS activity [39]. In detail, while in (c) and (a) plants the plastidial activity was almost undetectable, it increased proportionally to NO_3_^−^ availability in (n) and (na) roots, reaching the values of 0.0017 ± 0.0001 and 0.0007 ± 0.0001 μmol mg^−1^_prot_ min^−1^, respectively (Figure 6A). These results strongly support the idea that, in maize roots, GS2 is involved only in NO_3_^−^ assimilation and it is not influenced by NH_4_^+^ nutrition. Among the three identified GS2 forms, the GS2b is the only one of which the accumulation level was significantly increased in plants exposed to NO_3_^−^. This observation confirms our former proteomic analyses [32]. The comparison of GS2 accumulation levels (Figure 4A, B and C) and GS2 activity (Figure 6A) suggests the hypothesis that GS2b represented the form really participating in catalytic activity. Interestingly, this spot represented also the only phosphorylated GS2 (Figure 5).

The reciprocal position of the GS2 spots was comparable with those observed in the leaf maize proteome. Indeed, in leaves two GS2 spots were identified of which the accumulation levels were differently related to the enzymatic activity. In particular, the more acidic spot, matching with GS2b, was accumulated proportionally, while the basic one disappeared with the increment of the enzymatic activity [19]. The GS2c electrophoretic position (Figure 3) as well as its trend of accumulation (Figure 4) showed a high degree of similarity with this last observation. As a whole, the results suggest that GS2a and GS2c might be transitional forms of the enzyme, probably originated by PTMs. The chemical and/or immunological detection of sugar and nitric oxide moieties did not lead to any positive results (*i.e.* no signal on PVDF membranes corresponding to GS isoforms), but it is not possible to exclude the occurrence of several other PTMs.

### The responses to inorganic N sources of the GS1 isoforms in maize roots

According to the proposed model, the presence of phosphorylation on GS1 is associated with active enzymatic forms, where it promotes protection from degradation and the interaction with activating 14-3-3 proteins [20]. The evaluation that the spots 4, 5 and 6 were phosphorylated in all conditions tested (Figure 5) supports the hypothesis that they represented active subunits of the root GS1 enzyme.

Firstly, it is possible to note that all three GS1 spots were detected in (c) plants, in which spot 4 was predominant (1.14 ± 0.05 %*Vol*, Figure 4D, E and F). Their presence in roots of starved plants confirmed a GS1 involvement in the use and/or recycling of endogenous N reserves. Spot 4 was the least influenced by the plant nutritional status, as it only decreased by about 20% in response to NH_4_^+^ (Figure 4D). This trend was in agreement with previous transcriptional analyses highlighting that GS1-3 and GS1-4 mRNAs slightly decreased in response to N as well as with the unchanged intensity of GS1 band in SDS-PAGE (Figure 2B; [23,24]). Together with the fact that in maize *gln1-3* and *gln1-4* mutants the vegetative biomass is not affected [19], these results reinforce the hypothesis that other root isoforms are able to sustain N assimilation during vegetative growth.

Interestingly, spots 5 and 6 showed marked and different changes (Figure 4E and F). The GS1-1 isoform specifically increased in response to NH_4_^+^ nutrition, becoming the most abundant one in (a) roots with the highest measured value (1.78 ± 0.24 %*Vol*). It is worth noting that the changes in GS1-1 accumulation reflected the NH_4_^+^ availability, reaching an increment of about +112% and +54% in (a) and (na) plants, respectively. The responses of GS1-5 isoform attested an even higher increase of about +222% in (a) condition. Likewise, in (a) plants the GS synthetase activity significantly increased (Figure 6B). These results confirm that NH_4_^+^ induces GSr (Figure 2B, [24]) and, for the first time, they allowed us to discern the differential contribution of its components. Moreover, GS1-5 showed a peculiar doubling (+86%) in (n) plants. The fact that GS1-5 is metabolically active in (n) roots is reinforced by the estimation that its change is the only one to be associated with the increase in total GS synthetase activity in the (n) condition (Figure 6B). Interestingly, these observations confirm at protein and enzymatic order the induction of GS1-5 transcript by NO_3_^−^ exposure recently observed in the T250 line [40]. Taken together, these results provide the first information about the functional role of the GS1-5 isoform, providing evidence of its involvement in root NO_3_^−^ metabolisation.

The GS synthetase activity in the total protein fraction, starting from 0.071 ± 0.007 μmol mg^−1^_prot_ min^−1^ in starved plants (c), increased to a similar extent in N treated ones (Figure 6B). This feature suggests that GS1 followed a saturation kinetic, probably because the elevated concentrations of NO_3_^−^ and/or NH_4_^+^ overfilled the metabolic capability of the maize root organ. However, the root metabolic capability seemed to be sustained by different GS1 isoforms, according to different mineral N sources. This was particular evident in (na) roots where GS1-1, GS1-5 and GS2 were accumulated to an intermediate level (Figure 4). In order to get better information about this aspect, the percentage of thermal inactivation of total GS transferase activity, induced by 45°C for 10 min, was evaluated (Figure 6C). According to the physicochemical characterization, the Isoleucine-161 in the GS1-4 sequence confers thermal stability, while the substitution with Ala-161 clearly renders the GS1-1 more heat-labile than GS1-4 [15,24]. Considering that GS1-4 and GS1-1 shared this feature with GS1-3 and GS1-5 respectively, it is possible to assume that the thermal inactivation measured reflected the proportion of GS activity ascribable to the GS1-1 with GS1-5 subunits. Such evaluation allowed us to confirm that GS1-1 and GS1-5 were fundamental for the assembling of GS active enzyme in (c) and (n) roots, where the thermal treatment provoked a loss in activity of about 97% and 98%, respectively. It is also worth noting how the contribution of GS1-3 and GS1-4 gained in importance in roots of plants exposed to NH_4_^+^, especially if in co-provision with NO_3_^−^ (Figure 6C).

Overall, this proteomic investigation confirmed the GS1 involvement in N recycling as well as in the root assimilation of NO_3_^−^ and NH_4_^+^, and at the same time, it allowed us to propose that specific and combined isoforms sustain these different metabolic tasks.

### The inorganic N sources differently affected the amino acid accumulation and composition in root, leaf and xylem sap

**Figure 7:**
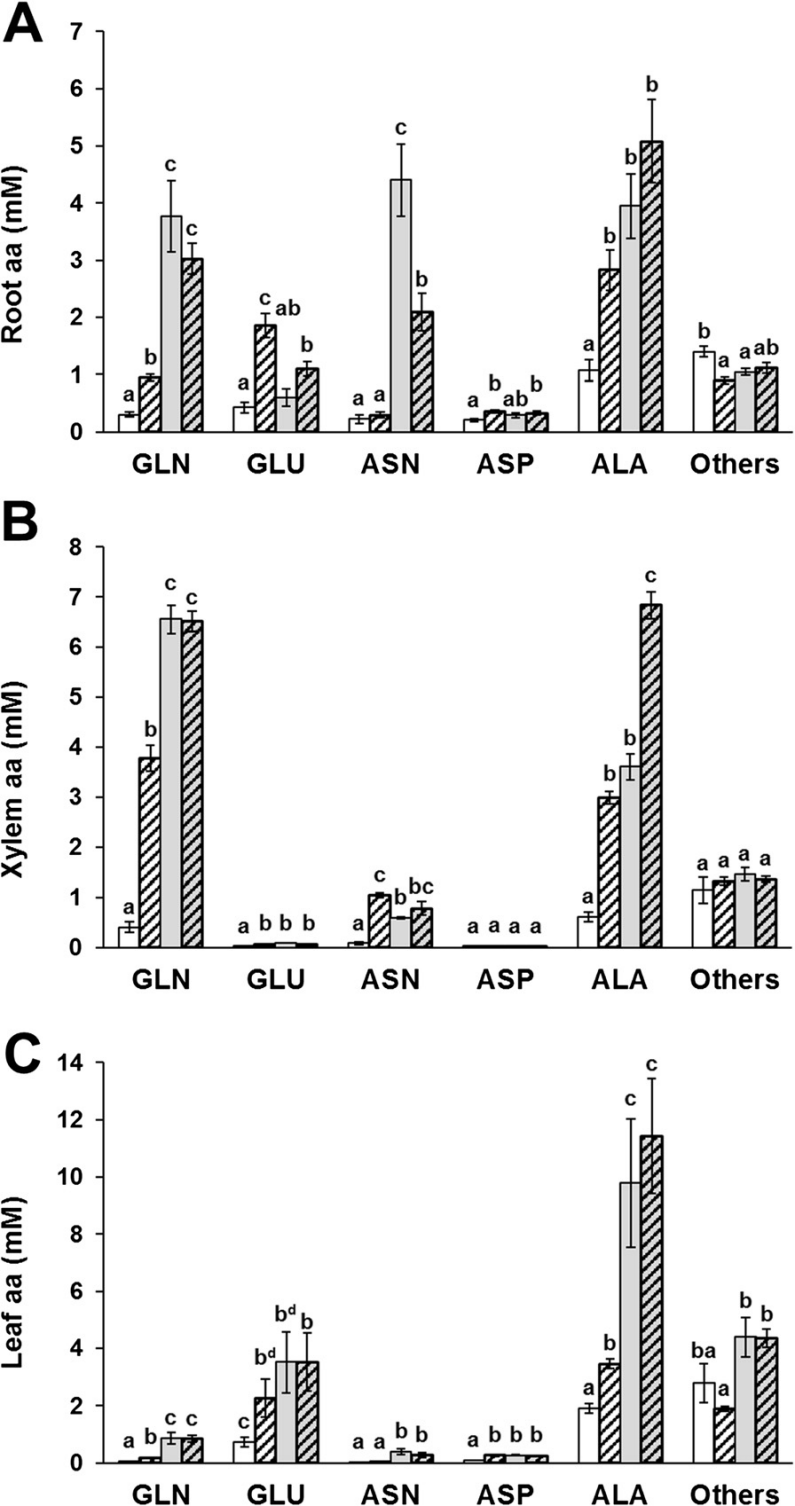
**Levels of amino acids in maize plants in response to inorganic N sources.** Concentration of the main amino acids in roots **(A)**, xylem sap **(B)** and leaves **(C)** in maize plants grown for 10 days without N sources and then exposed for the last 30 h to absence of N (c, white bars), to 10 mM NO_3_^−^ (n, crossed white bars), to 10 mM NH_4_^+^ (a, grey bars) or to 5 mM NO_3_^−^ + 5 mM NH_4_^+^ (na, crossed and grey bars). Graphs show the amino acid concentrations (mM) as average values ± SE (roots and leaves n = 8, xylem sap n = 6). The letters above are assigned according to Student’s t-test (p ≤ 0.01; ^d^ p ≤ 0.05). Detailed quantification of individual amino acids in root, xylem and leaf is reported in Additional file 1: Table A8, A9 and A10, respectively.

In order to appreciate the extent by which the GS activation induced by the experimental treatments affected the plant N metabolism, the composition of amino acids in different tissues was measured. Figure 7 points out the changes related to the mostly more abundant amino acids, gathering the others in a single group, detected in roots (Figure 7A), in xylem sap (Figure 7B) and in leaves (Figure 7C) of plants exposed to the four N conditions. The comprehensive amino acid compositions of the three tissues are detailed in the Additional file 1: Tables A8, A9 and A10, respectively.

The provision of N provoked a significant increase in amino acid level at whole plant scale, but the extent of these increments varied in relation to the inorganic N source and/or organ. The total amino acids concentration in roots and leaves of (a) plants was almost double the amounts in (n) plants, confirming that NH_4_^+^ nutrition promoted a more intensive N assimilation than NO_3_^−^ (Figure 7; [4]). This aspect was mirrored by the total protein amounts in the root systems (Additional file 1: Figure A4). Because the total amino acid concentration reached similar values both in roots and leaves during the (a) and (na) treatments (Additional file 1: Tables A8 and A10), the T250 line did not show a marked synergistic response to the co-provision of NO_3_^−^ with NH_4_^+^.

Gln was the amino acid subjected to the greatest and most prevalent changes after the exposure of the plants to N as well as being the main compound for N translocation in the xylem sap, in which it reached the maximum value of 6.55 ± 0.28 mM in (a) plants (Figure 7B). Moreover, all three organs showed high amounts of Ala, which represented up to 40%, 44% and 55% of the total amino acids in roots, xylem sap and leaves, respectively (Additional file 1: Tables A8, A9 and A10). Considering that previous studies on maize plants grown in high N reported Ala percentages in xylem and leaves ranging from 5% to 20-29%, respectively [19,35], the higher percentage of Ala may be a peculiarity of the T250 line. Taking into account the involvement of Ala and aspartic acid (Asp) in the C4 photosynthesis, it is also possible that this variability derives from differences in the times of the day when leaves were sampled as well as from differences in leaf developmental stage and in plant N regime [35,41].

Looking at the root system, the Gln level starting from 0.30 ± 0.04 mM in (c) condition increased to 0.94 ± 0.06 mM in (n) plants but it reached the higher values of 3.77 ± 0.62 mM and 3.02 ± 0.27 mM in (a) and (na) plants, respectively (Figure 7A). This trend was associated with comparable upsurges in the xylem sap (Figure 7B). Since the root GS synthetase activity was very similar in all three N treatments (Figure 6B), it is reasonable to suppose that the lowest level of Gln in (n) roots did not result from an enzymatic control on GS but rather from a metabolic regulation on NO_3_^−^ reduction steps, limiting the free NH_4_^+^ in the cell. The slower N assimilation in (n) roots was associated with an accumulation of Glu and Asp (Figure 7A), supporting the idea that the GS/GOGAT and the Tricarboxylic Acid (TCA) cycles were reciprocally balanced, sufficiently to sustain the storage of intermediates. In addition, the similar Gln concentrations observed in roots and xylem saps of (a) and (na) plants (Figure 7A and B) indicated that the maximum capacity of plants to synthesize and translocate Gln was already reached during the exposure to the lowest availability of NH_4_^+^ (*i.e.* 5 mM). This is also consistent with the saturation kinetic of the GS synthetase activity described above (Figure 6B).

Otherwise, Asn significantly incremented only in roots exposed to NH_4_^+^, reaching the values of 4.40 ± 0.63 mM and 2.09 ± 0.32 mM in (a) and (na) conditions, respectively (Figure 7A). Asn represents one of the main compounds for N storage and transport due to its high N/C ratio and stability. It is synthesized by Asn synthetase (AS, EC6.3.5.4) by the amidation of Asp using Gln as amino donor, but several studies have indicated an NH_4_^+^-dependent synthetase activity in plants ([42] and references therein). The Asn changes are in agreement with the observation that in maize roots the AS gene expression is influenced by C/N ratio since it is induced by carbohydrate limitation and by supplies of NH_4_^+^, Gln, Asn, Asp but not of Glu [43]. Moreover, in maize mutants deficient for GS1-3 and/or GS1-4 a higher leaf content of Asn and Ala compared with than of the wild-type was reported, suggesting compensatory involvement in NH_4_^+^ (re)assimilation [44]. In this work, the levels of Asn in root tissues were quite proportional to the root NH_4_^+^ concentrations, but not to the Gln ones, suggesting that the Asn accumulation could be involved in a mechanism of cell protection from high NH_4_^+^, which appeared specifically induced by the cation and not by Gln. This induction was not associated with a comparable upsurge of Asn translocation. On the contrary, the Ala concentration strongly increased in all three organs in response of N availability (Figure 7). The highest increment of Ala translocation was observed during the (na) treatment, showing a synergistic trait related to co-provision of NO_3_^−^ with NH_4_^+^. Recently it was proposed that Ala and pyruvate translocations might have important roles for the maintenance of C/N balance throughout plants [45]. Considering that in leaves the Asn re-assimilation necessarily releases free NH_4_^+^ [46], the sequestration of Asn in roots and the preferential translocation of Gln and Ala might participate in the mechanism of NH_4_^+^ tolerance observed in T250.

The Glu levels showed marked differences between root and leaf systems under the same nutritional treatment. In roots, Glu accumulation was increased in the presence of NO_3_^−^, reaching the values of 1.10 ± 0.13 mM and 1.86 ± 0.21 mM in (na) and (n) plants, respectively. However, the provision of NH_4_^+^ (a) was associated with a very low level of 0.60 ± 0.16 mM, comparable with the (c) plants (Figure 7A). Glu was scarcely translocated in the xylem sap, while in leaves the N availability sustained a generalized increment of Glu that mirrored the total amino acid concentrations (Figure 7B and C). These observations suggest that the balancing of the GS/GOGAT cycle distinctly diverged between the two organs. In particular, in leaf the high amount of Gln received from the xylem sap appeared re-assimilated to restore Glu. On the contrary, in the root system the NH_4_^+^ exposure seemed to hamper the accumulation of Glu, resulting in a large prevalence of Gln. Several studies have given evidence of the existence of a Glu homeostasis in plants, probably involved in plant C/N perception, which is perturbed by NADH (reduced Nicotinamide Adenine Dinucleotide) and 2OG (2-oxoglutarate) availability [6,9,47]. Hence, it is possible to suppose that the peculiar shortage of Glu in (a) roots was associated with a scarce provision of 2OG by the TCA cycle to the GS/GOGAT system. The involvement of the TCA cycle also seems to be supported by the concerted changes of Asp (Figure 7). Considering that the (a) and (na) roots showed similar extent of N assimilation as well as that the (a) roots were characterized by the highest content of reducing sugars, it is unlikely that this imbalance derived from a lack of C skeletons. Instead, it is more conceivable that the lack of Glu was related to an excess of reducing power. In fact, the high N assimilation in roots exposed to NH_4_^+^ could be associated with a strong activation of anaplerotic reactions for C skeletons, leading to a production of NADH exceeding the metabolic requests. Considering that this excess could cause a feedback inhibition on the TCA cycle [48] it is possible that the outcome could be a very low availability of 2OG in the cell. This consideration is consistent with the evidence that in roots of *Arabidopsis thaliana* the supply of NH_4_^+^ compared to NO_3_^−^ promotes a higher capacity of respiratory bypass pathways involved in the dissipation of excess of redox equivalents [49]. In addition, the co-provision of NO_3_^−^ with NH_4_^+^ sustained a higher accumulation of Glu in (na) roots (Figure 7A). This could be a synergistic effect by which, even if the N assimilation in (a) and (na) roots was similar, the consumption of reducing power by NO_3_^−^ reducing steps could be associated with a minor inhibition of the TCA cycle. It is interesting to note that, because the Asn and Ala are synthesized by the transfer of the amino group of Gln on C skeletons (*i.e.* pyruvate and oxaloacetate) available out of the TCA cycle [6], their synthesis could contribute towards regenerating Glu with a NADH production lower than the GS/GOGAT route.

Overall, the analysis of the amino acid composition confirms the activation of GS observed by the proteomic and enzymatic approaches, highlighting the relevance of root responses in the N economy. Moreover, the results provide new information about the metabolic regulation of the GS/GOGAT cycle that seem to be deeply influenced by several aspects, such substrates and coenzymes, as well as by the biosynthetic pathways of other amino acids.

## Conclusion

Taken together, the results give novel insights about the multiplicity of factors involved in GS regulation. Firstly, the work provides new evidence that in maize different GS isoenzymes/isoforms have distinct metabolic functions, diverging between root and leaf system. Interestingly, the proteomic discrimination of the GS1 proteins revealed that in roots the cytosolic enzyme also contributes in NO_3_^−^ assimilation by GS1-5 activity, providing first indications about the role of this isoform. At the same time, the changes in enzymatic properties as well as the presence of phosphorylation confirmed the involvement of PTMs. It is conceivable that these observations may be useful for future studies aimed to investigate the rearrangement of GS native enzyme and its interaction with regulatory proteins. Furthermore, the analyses of amino acid composition in roots, xylem sap and leaves provides novel information about the fact that in roots the GS/GOGAT cycle was not only regulated at molecular level but it was also deeply influenced by biochemical factors, like substrates and cell redox status. Finally, from a physiological point of view, it is interesting to note that the work gives new insights about the relevance of Glu, Asn and Ala in plant C/N balance in response to nitrate and/or ammonia nutritions.

## Methods

### Plant materials

Maize seeds of the T250 inbred line, kindly provided by Prof. Zeno Varanini of the University of Verona, Italy, were germinated in the dark at 26°C for 72 h. The seedlings were transferred to a hydroponic system in a growth chamber with a photoperiod of 16/8 h at 26/22°C, assuring PPFD of 200 μmol m^−2^ s^−1^ and at constant relative humidity of 65%. After incubation in 4 mM CaSO_4_ for 48 h, the plants were grown for the following eight days in a solution of 400 μM CaSO_4_, 200 μM K_2_SO_4_, 175 μM KH_2_PO_4_, 100 μM MgSO_4_, 20 μM Fe-EDTA, 5 μM KCl, 2.5 μM H_3_BO_3_, 0.2 μM CuSO_4_, 0.2 μM ZnSO_4_, 0.2 μM MnSO_4_, 0.05 μM Na_2_MoO_4_, pH = 6.1. All hydroponic solutions were continuously aerated and renewed every three days. After this period of N starvation, at the beginning of the light period plants were transferred for 30 h into fresh growing solutions of the following four treatments, balanced with K_2_SO_4_: i) N absence (c); ii) 10 mM NO_3_^−^ (n); iii) 10 mM NH_4_^+^ (a); iv) 5 mM NO_3_^−^ + 5 mM NH_4_^+^ (na). (For details, see Additional file 1: Figure A1 and Table A1). At the time of sampling, roots and leaves were separately collected, frozen in liquid N_2_ and stored at −80°C. The root systems of plants destined for NO_3_^−^ and NH_4_^+^determination were rinsed in aerated ice-cold solution (5 mM K_2_SO_4_, 0.4 mM CaSO_4_) in the growth chamber for 15 min before sampling. For xylem sap collection, the plants were maintained in the hydroponic solution and de-topped by cutting the stem with a razor blade just above the first internode. The cut surface was rinsed twice with distilled water and blotted with paper. Then the stem was encircled with a silicon tube and the liquid drawn in the first 5 min was discarded. Finally, the xylem saps collected from 5 to 25 min from six plants were pooled into a biological sample, weighed and stored at −80°C.

### Determination of nitrate, ammonium, reducing sugars and sucrose

For NO_3_^−^ and sugar content determination, organ samples were treated as described by Prinsi and co-workers [32]. NO_3_^−^ and sugars were quantified according to Cataldo *et al*. [50] and Nelson [51], respectively. NH_4_^+^ was extracted from roots and leaves by adding 3% (w/w) of polyvinylpolypyrrolidone (PVPP), homogenizing in 4 vol of 50 mM Tris–HCl pH 7.4, 10 mM imidazole, 10 mM ascorbic acid, 0.5% (v/v) β-mercaptoethanol in ice and then centrifuging at 10,000 *g* for 20 min at 4°C. NH_4_^+^ concentration was determined by the Ammonia Assay Kit (Sigma-Aldrich) according to manufacturer’s instructions. For NO_3_^−^ and NH_4_^+^ detection leaf and root samples were filtered by Millipore Millex HV cartridges (0.45 μm) while the xylem saps were directly analysed. All of the three analyses were conducted on three biological samples, each composed by three plants (and six for xylem sap), analysed in duplicate (n = 6).

### Extraction of soluble protein fraction and electrophoretic analyses

The leaves or roots collected from 18 plants were pooled into one sample used for the further analysis as one independent biological replicate, powdered in liquid N_2_ and stored at −80°C. The soluble protein fraction (*i.e.* whole proteome depleted of membrane proteins) was separated by centrifugation at 100,000 *g* at 4°C for 38 min and then the protein components were purified by consecutive precipitations in 0.1 M ammonium acetate in methanol and acetone as described by Prinsi and co-workers [32], optimizing the procedure by the addition of phosphatase inhibitors in the extraction buffer (10 mM NaF, 1 mM Na_3_VO_4_). 1D-PAGE was conducted on 10% acrylamide gel [52]. The 2D-PAGE were done according to Prinsi and co-workers [32] but adapting the isoelectric focusing on pH 4–7, 13 cm IPG strips (GE Healthcare) for a total of 25 kV and the SDS-PAGE into 10% acrylamide gels. The qualitative investigations by Western Blot (WB) was conducted as described by Bernardo and co-workers [53] both on 1D and 2D profiles on two biological replicates analyzed in duplicate (n = 4), using separately two primary antibodies: against nitrate reductase (NR, EC1.7.99.4, 1:1000, Agrisera AS08310, polyclonal [54]) and against all GS (1:10,000, GS1 + GS2 global antibody, Agrisera AS08295, polyclonal with reactivity in maize proved in producer’s technical sheet and in Arabidopsis by [55]). The WB were visualized by anti-rabbit IgG conjugated with alkaline phosphatase. For the 2D approach, the map sector corresponding to p*I* 4–6.5 and MW 32–55 kDa was blotted and, before the blocking procedure, the filters were reversibly stained with Ponceau S 0.5% (w/v) in 1% (v/v) acetic acid in order to orientate the pattern. For the qualitative determination of GS phosphorylation, we employed Sypro Ruby® coupled with Pro-Q® Diamond phosphoprotein gel stain (Molecular Probes) according to the manufacturer’s instructions (n = 4). For the quantitative analysis, three biological replicates in duplicate (n = 6) were stained with colloidal Coomassie Brilliant Blue G-250 (cCBB; [56]) and analyzed with ImageMaster 2-D Platinum Software (GE Healthcare) in order to quantify the spots (*%Vol:* percentage of the total spot volume) assigned to GS by WB.

### Protein identification by LC-nESI-MS/MS

The six spots visualized by WB against GS were excised from cCBB 2D gels independently for each experimental condition, obtaining 24 samples. After trypsin digestion [32], the samples were analyzed by a 6520 Q-TOF mass spectrometer with HPLC Chip Cube source driven by 1200 series nano/capillary LC system (Agilent Technologies). The nLC separation was done on 75 μm x 43-mm column (Zorbax SB, C18, 300 Å), applying a 13-min Acetonitrile (ACN) gradient (from 5% to 60% v/v) in 0.1% (v/v) formic acid at 0.4 μl min^−1^. The mass spectrometer ran in positive ion mode acquiring 4 MS spectra s^−1^ from 300 to 3000 m/z. The auto-MS/MS mode was applied from 50 to 3000 m/z with a maximum of 4 precursors per cycle and an active exclusion of 2 spectra for 0.1 min. Peptide identification was performed by Spectrum Mill MS Proteomics Workbench (Rev B.04.00.127; Agilent Technologies). Cysteine carbamidomethylation and methionine oxidation were set as fixed and variable modifications, respectively, accepting two missed cleavages per peptide. The search was conducted against the subset of *Zea mays* protein sequences (Oct 2013, 172261 *entries*) downloaded from the National Center for Biotechnology Information [57] and concatenated with the reverse one. The threshold used for peptide identification was Spectrum Mill score ≥ 9, Score Peak Intensity ≥ 70%, mass MH^+^ Error ≤ 10 ppm, Local False Discovery Rate ≤ 0.1% and Database Fwd-Rev Score ≥ 2. Each sample was analyzed in triplicate and independently assigned to a GS isoform, according to the discriminating peptides. For each spot position (n. 1 to 6 in Figure 3A), the isoform assignment was independently confirmed among all the four 2D profiles corresponding to the four nutritional conditions. Then, to provide the overall information obtained about protein sequencing, MS data regarding each isoform were summed. Physical properties of the isoforms were predicted by *in silico* tools at ExPASy [58].

### Determination of GS activity

For the determination of total GS activity, frozen root systems were powdered in liquid N_2_ to which was added 0.5% (w/w) PVPP, and extracted in 3 volumes of 50 mM Tris–HCl pH = 7.8, 10 mM MgSO_4_, 1 mM dithiothreitol, 10% (v/v) ethylene glycol. The samples were centrifuged at 12,000 *g* for 20 min at 4°C and filtered on G-25 columns (PD-10, GE Healthcare), eluting in 25 mM Tris–HCl pH = 7.8, 10 mM MgSO_4_. Root plastids were isolated according to Redinbaugh and Campbell [59]. The GS synthetase and transferase activities were measured by the spectrophotometric determination of γ-glutamylhydroxamate, as described by Lea *et al*. [60] and Cullimore and Sims [61], respectively. The transferase activities were measured prior to and after thermic treatments at 45°C for 10 min. Protein contents were quantified by the BioRad protein assay. All experiments were replicated on three independent biological samples, each derived from six plants (n = 3).

### Determination of amino acid composition by LC-ESI-MS analysis

The frozen root and leaf samples, each collected from six plants, were homogenized in 3 volumes of 1 mM tridecafluoroheptanoic acid (TDFHA), 50% (v/v) methanol. Samples were shaken for 10 min at 4°C and then centrifuged twice at 14,000 *g* for 20 min at 4°C. The underivatized supernatants and the collected xylem saps were finally diluted to 0.5 mM TDFHA, 25% (v/v) methanol. The LC-ESI-MS analyses were conducted by an Agilent Technologies 1200 Series capillary pump coupled with dual ESI source on 6520 Q-TOF mass spectrometer according to Armstrong *et al*. [62]. Briefly, LC runs were done on an XDB-C18 column (2.1 x 50 mm, 1.8 μm, Agilent Technologies) applying a 30 min non-linear gradient of 0.5 mM TDFHA/ACN with a flow rate of 200 μl min^-1^. The ESI source was set at 350°C, 3500 V and the fragmentor at 100 V. The data acquisition range was 50–350 m/z at 0.93 scans s^-1^. The quantitation was conducted on EIC for single MH^+^ in ±0.02 m/z window, accepting a mass error of ±5 mDa in ion identification and referring to calibration curves (Additional file 1: Table A11). The analyses were conducted on four root and leaf samples and three xylem sap samples analysed in duplicate (n = 8, n = 6).

## Additional file

Additional file 1:
**A single .pdf file containing.** **Figure A1.** Experimental design. **Figure A2.**1D-electrophoretic profiles of leaf protein samples and mass spectrometry characterization of the most prominent band. **Figure A3.** 2D-electrophoretic reproducibility of maize root protein samples. **Figure A4.** Soluble protein contents in maize roots. **Table A1.** Composition of the hydroponic solutions used for the 30 h nutritional treatments. **Table A2, A3, A4, A5, A6** and **A7.** LC-nESI-MS/MS characterization of spots n.1, 2, 3, 4, 5 and 6. **Table A8.** Levels of amino acids in roots. **Table A9.** Levels of amino acids in xylem sap. **Table A10.** Levels of amino acids in leaves. **Table A11.** Experimental parameters used for amino acid quantitation.

## Abbreviations

1D: One-dimensional
2D: Two-dimensional
2OG: 2-oxoglutarate
(a): Plants exposed to 10 mM NH_4_^+^
ACN: Acetonitrile
Ala: Alanine
AS: Asparagine synthetase
Asn: Asparagine
Asp: Aspartic acid
C: carbon
(c): control plants exposed to N absence
cCBB: colloidal Coomassie Brilliant Blue G-250
Glc: Glucose
Gln: Glutamine
Glu: Glutamic acid
GOGAT: Glutamate synthase
GS: Glutamine synthetase
LC-nESI-MS/MS: Liquid Chromatography-nanoElectroSpray Ionization-Tandem Mass Spectrometry
LC-ESI-MS: Liquid Chromatography-ElectroSpray Ionization-Mass Spectrometry
N: Nitrogen
(n): plants exposed to 10 mM NO_3_^−^
(na): Plants exposed to 5 mM NO_3_^−^ + 5 mM NH_4_^+^
NADH: Reduced Nicotinamide Adenine Dinucleotide
NH_4_^+^: Ammonium
NO_3_^−^: Nitrate
NR: Nitrate Reductase
PTMs: Post transcriptional/translational modifications
PVPP: Polyvinylpolypyrrolidone
SDS-PAGE: Sodium Dodecyl Sulphate-PolyAcrylamide Gel Electrophoresis
TCA cycle: Tricarboxylic acid cycle
TDFHA: Tridecafluoroheptanoic acid
WB: Western Blotting
