# Supplementary material for: Mineral nitrogen sources differently affect root glutamine synthetase isoforms and amino acid balance among organs in maize
Source: BMC Plant Biol. 2015 Apr 3;15:96. doi: 10.1186/s12870-015-0482-9 (PMC4393875; doi:10.1186/s12870-015-0482-9)
Supplement: Additional file 1: — A single .pdf file containing. Figure A1. Experimental design. Figure A2.1D-electrophoretic profiles of leaf protein samples and mass spectrometry characterization of the most prominent band. Figure A3. 2D-electrophoretic reproducibility of maize root protein samples. Figure A4. Soluble protein contents in maize roots. Table A1. Composition of the hydroponic solutions used for the 30 h nutritional treatments. Table A2, A3, A4, A5, A6 and A7. LC-nESI-MS/MS characterization of spots n.1, 2, 3, 4, 5 and 6. Table A8. Levels of amino acids in roots. Table A9. Levels of amino acids in xylem sap. Table A10. Levels of amino acids in leaves. Table A11. Experimental parameters used for amino acid quantitation. [file 12870_2015_482_MOESM1_ESM.pdf]

ADDITIONAL FILE 1

Additional material of the manuscript "Mineral nitrogen sources differently affect root glutamine synthetase isoforms and amino acid balance among organs in maize"

Bhakti Prinsi and Luca Espen

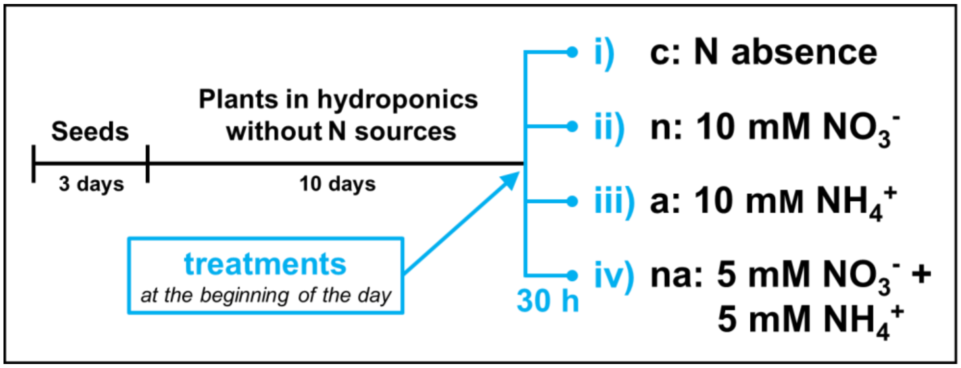

Additional Figure A1. Experimental design. T-250 maize seeds germinated in the dark. After 3 days, the seedlings were grown in a hydroponic system for 10 days in absence of N. Afterwards the plants were incubated for further 30 h in four different conditions: i) N absence (c); ii) 10 mM NO_3^- (n); iii) 10 mM NH_4^+ (a); iv) 5 mM NO_3^-+5 mM NH_4^+ (na). For details, see the materials and methods section and Additional Table 1.

Additional Table A1. Composition of the hydroponic solutions used for the 30 h treatments. Each solution was composed of 400 µM $CaSO_4$, 175 µM KH_2PO_4 , 100 µM $MgSO_4$, 20 µM Fe-EDTA, 5 µM KCl, 2.5 µM H_3BO_3, 0.2 µM $CuSO_4$, 0.2 µM $ZnSO_4$, 0.2 µM $MnSO_4$, 0.05 µM Na_2MoO_4 added according to the following treatments and adjusted to a pH=6.1.

Abbreviation	Condition	Specific composition	
**c**	Control in nitrogen absence	K_2SO_4	5 mM
**n**	10 mM NO_3^-	KNO_3	10 mM
**a**	10 mM NH_4^+	$(NH_4)_2SO_4$	5 mM
		K_2SO_4	5 mM
**na**	5 mM NO_3^-+5 mM NH_4^+	KNO_3	5 mM
		$(NH_4)_2SO_4$	2.5 mM
		K_2SO_4	2.5 mM

Additional Figure A2. **1D-electrophoretic profiles of leaf protein samples and mass spectrometry characterization of the most prominent band in the profiles.** Leaf samples from plants grown for 10 days without N sources and then exposed for the last 30 h to absence of N (c), to 10 mM NO_3^- (n), to 10 mM NH_4^+ (a) or to 5 mM NO_3^-+5 mM NH_4^+ (na) were analysed by 1D-SDS-PAGE. The bands in the dotted boxes were individually picked from the gel and analysed by LC-nESI-MS/MS. In red are reported the sequenced peptides. The data are the sums of all the analyses reporting the unique peptides with the best score assignment. **n**: spectrum number; **%f**: frequency of identification of the peptides among all the analyses; **z:** charge state; **Local FDR (%)**: Local False Discovery Rate (%); **SPI (%):** Score Peak Intensity (%); **Sequence map**: forward-slashes for locations of y-ions, backslashes for locations of b-ions, vertical lines for locations of both b- and y-ions. The abundance (%) was evaluated as the percentage of the sum of spectra related to pyruvate orthophosphate dikinase respect the total spectra intensity of the band. No peptide related with nitrate reductase was ever detected.

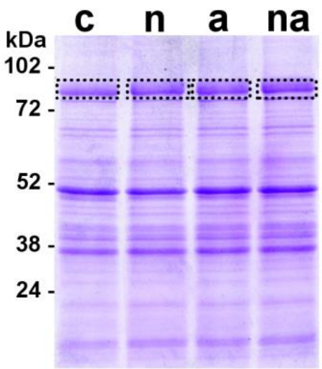

>gi|168586|gb|AAA33498.1| pyruvate,orthophosphate dikinase [Zea mays]

MTASVSRAICVQKPGSKCTRDREATSFARRSVAAPRPPHAKAAGVIRSDSGAGRGQHCSPLRAVVDAAPIQT
TKKRVFHFGKGKSEGNKTMKELLGGKGANLAEMASIGLSVPPGFTVSTEACQQYQDAGCALPAGLWAEIVD
GLQWVEEYMGATLGDPQRPLLLSVRSGAAVSMPGMMDTVLNLGLNDEVAAGLAAKSGERFAYDSFRRFLD
MFGNVVMDIPRSLFEEKLEHMKESKGLKNDTDLTASDLKELVGQYKEVYLSAKGEPFPSDPKKQLELAVLAVF
NSWESPRAKKYRSINQITGLRGTAVNVQCMVFGNMGNTSGTGVLFTRNPNTGEKKLYGEFLVNAQGEDVVA
GIRTPEDLDAMKNLMPQAYDELVENCNILESHYKEMQDIEFTVQENRLWMLQCRTGKRTGKSAVKIAVDMVN
EGLVEPRSAIKMVEPGHLDQLLHPQFENPSAYKDQVIATGLPASPGAAVGQVVFTAEDAEAWHSQGKAAILV
RAETSPEDVGGMHAAVGILTERGGMTSHAAVVARGWGKCCVSGCSGIRVNDAEKLVTIGGHVLREGEWLSL
NGSTGEVILGKQPLSPPALSGDLGTFMAWVDDVRKLKVLANADTPDDALTARNNGAQGIGLCRTEHMFFASD
ERIKAVRQMIMAPTLELRQQALDRLLPYQRSDFEGIFRAMDGLPVTIRLLDPPLHEFLPEGNIEDIVSELCAETG
ANQEDALARIEKLSEVNPMLGFRGCRLGISYPELTEMQARAIFEAAIAMTNQGVQVFPEIMVPLVGTPQELGH
QVTLIRQVAEKVFANVGKTIGYKVGTMIEIPRAALVADEIAEQAEFFSFGTNDLTQMTFGYSRDDVGKFIPVYLA
QGILQHDPFEVLDQRGVGELVKLATERGRKARPNLKVGICGEHGGEPSSVAFFAKAGLDYVSCSPFRVPIARL
AAAQVLV

462/947 AA: 48.8%. Total peptide (unique): 149 (29). Reproducibility: 100%. Abundance (%) in 1D-SDS-PAGE band: 65.6% ± 2.8.

<table>
<tr><th>n</th><th>%f</th><th>z</th><th>Score</th><th>Local FDR (%)</th><th>DB FWd-Rev Score</th><th>SPI (%)</th><th>Sequence map</th><th>MH+ Matched (Da)</th><th>MH+ Mass Shift (Da)</th><th>MH+ Error (ppm)</th></tr>
<tr><td>1</td><td>1/4</td><td>3</td><td>15.35</td><td><0.1%</td><td>15.35</td><td>85.7</td><td>(R)AALV\A\DEIAE\QAE\FFSF/GTNDL/T/Q/M/T/F/GY/SR(D)</td><td>3429.594</td><td>-0.0049</td><td>-1.4</td></tr>
<tr><td>2</td><td>3/4</td><td>2</td><td>23.26</td><td><0.1%</td><td>23.26</td><td>90.9</td><td>(R)A E|T/S/P E/D|V|G/G M H/A|A/V|G I/L\T E/R(G)</td><td>2140.034</td><td>0.0074</td><td>3.5</td></tr>
<tr><td>3</td><td>4/4</td><td>2</td><td>20.26</td><td><0.1%</td><td>20.26</td><td>94</td><td>(K)A G/L|D|Y|V|S|C/S/P F/R(V)</td><td>1371.636</td><td>0.0036</td><td>2.6</td></tr>
<tr><td>4</td><td>2/4</td><td>2</td><td>17.72</td><td><0.1%</td><td>12.22</td><td>84.5</td><td>(R)A/m|D|G|L|P V|T/I/R(L)</td><td>1072.582</td><td>15.9988</td><td>3.6</td></tr>
<tr><td>5</td><td>1/4</td><td>2</td><td>17.11</td><td><0.1%</td><td>17.11</td><td>73.5</td><td>(K)C/C|V|S|G/C/S/G I/R(V)</td><td>1155.47</td><td>0.0106</td><td>9.1</td></tr>
<tr><td>6</td><td>3/4</td><td>2</td><td>24.37</td><td><0.1%</td><td>24.37</td><td>98.2</td><td>(R)E G E|W|L|S|L|N/G/S T/G E/V/I/L/G K(Q)</td><td>1888.965</td><td>-0.0028</td><td>-1.5</td></tr>
<tr><td>7</td><td>4/4</td><td>2</td><td>21.63</td><td><0.1%</td><td>21.63</td><td>98</td><td>(K)E M/Q|D|I|E/F/T/V/Q/E/N R(L)</td><td>1638.743</td><td>0.0037</td><td>2.3</td></tr>
<tr><td></td><td>4/4</td><td>2</td><td>21.49</td><td><0.1%</td><td>21.49</td><td>93</td><td>(K)E m|Q/D/I|E|F/T/V/Q/E/N/R(L)</td><td>1638.743</td><td>15.9977</td><td>1.7</td></tr>
<tr><td>8</td><td>1/4</td><td>2</td><td>16.87</td><td><0.1%</td><td>16.87</td><td>71</td><td>(K)F I/P V Y\L\A Q\G I|L/Q/H|D|P F E/V/L/D|Q R(G)</td><td>2598.372</td><td>0.002</td><td>0.8</td></tr>
<tr><td>9</td><td>4/4</td><td>2</td><td>22.03</td><td><0.1%</td><td>22.03</td><td>97.2</td><td>(R)F L|D|M|F|G/N V|V|M/D/I|P R(S)</td><td>1653.813</td><td>0.0086</td><td>5.2</td></tr>
<tr><td></td><td>4/4</td><td>2</td><td>23.14</td><td><0.1%</td><td>23.14</td><td>94.8</td><td>(R)F L|D|M|F|G/N/V|V/m/D/I|P R(S)</td><td>1653.813</td><td>15.9984</td><td>2.1</td></tr>
<tr><td></td><td>4/4</td><td>2</td><td>25.13</td><td><0.1%</td><td>25.13</td><td>97.9</td><td>(R)F L|D|m|F/G/N V|V|M/D/I|P/R(S)</td><td>1653.813</td><td>16.0014</td><td>3.9</td></tr>
<tr><td></td><td>4/4</td><td>2</td><td>23.01</td><td><0.1%</td><td>23.01</td><td>87.8</td><td>(R)F L|D|m|F|G/N V|V|m/D/I|P R(S)</td><td>1653.813</td><td>31.9908</td><td>0.6</td></tr>
<tr><td>10</td><td>4/4</td><td>2</td><td>22.2</td><td><0.1%</td><td>22.2</td><td>95</td><td>(K)G/L/K|N|D|T/D L/T|A/S/D|L/K(E)</td><td>1490.77</td><td>0.0038</td><td>2.6</td></tr>
<tr><td>11</td><td>4/4</td><td>2</td><td>26.74</td><td><0.1%</td><td>26.74</td><td>97.3</td><td>(R)GTAVNV|Q/C|M/V|F/G/NM/GN/T/S/GT/G\V/L/F/T/R(N)</td><td>2718.28</td><td>0.0051</td><td>1.9</td></tr>
<tr><td></td><td>1/4</td><td>2</td><td>18.6</td><td><0.1%</td><td>18.6</td><td>87.1</td><td>(R)GTAVNV\Q\CM/V|F|GNmGN/T/SGT/GV/L/F/T/R(N)</td><td>2718.28</td><td>15.9931</td><td>-0.7</td></tr>
<tr><td></td><td>2/4</td><td>2</td><td>23.7</td><td><0.1%</td><td>23.7</td><td>86.2</td><td>(R)GTAV|NVQ/CmV\F|G/N/M|GN/T/S/GT/GV/L/F/T/R(N)</td><td>2718.28</td><td>15.9931</td><td>-0.7</td></tr>
<tr><td>12</td><td>4/4</td><td>2</td><td>23.38</td><td><0.1%</td><td>19.31</td><td>98.6</td><td>(K)I A|V|D\M|V|N/E|G/L|V|E|P R(S)</td><td>1541.799</td><td>0.0033</td><td>2.2</td></tr>
<tr><td></td><td>4/4</td><td>2</td><td>24.99</td><td><0.1%</td><td>21.72</td><td>96.1</td><td>(K)I/A|V/D\m|V|N/E|G/L|V|E|P R(S)</td><td>1541.799</td><td>16.0001</td><td>3.3</td></tr>
<tr><td>13</td><td>1/4</td><td>3</td><td>15.98</td><td><0.1%</td><td>15.98</td><td>93.6</td><td>(K)K Q L E L A V\L\A|V|F|N/S/W E/S/P R(A)</td><td>2087.128</td><td>0.0012</td><td>0.6</td></tr>
<tr><td>14</td><td>4/4</td><td>2</td><td>23.66</td><td><0.1%</td><td>19.89</td><td>96.7</td><td>(R)L G/I|S|Y|P/E/L/T/E/M/Q A R(A)</td><td>1607.81</td><td>0.0028</td><td>1.7</td></tr>
<tr><td></td><td>4/4</td><td>2</td><td>23.98</td><td><0.1%</td><td>18.75</td><td>92.5</td><td>(R)L G I|S|Y|P E/L/T/E/m/Q A/R(A)</td><td>1607.81</td><td>15.9994</td><td>2.7</td></tr>
<tr><td>15</td><td>1/4</td><td>3</td><td>21.36</td><td><0.1%</td><td>21.36</td><td>91.7</td><td>(R)LLDPPLHEFL\PEGNIED\IV|SE|L|C/A/E/T/G/A/N/QED/AL/A/R(I)</td><td>3975.928</td><td>-0.0032</td><td>-0.8</td></tr>
<tr><td>16</td><td>4/4</td><td>2</td><td>23.25</td><td><0.1%</td><td>15.54</td><td>100</td><td>(K)L/S|E|V|N|P M/L/G F/R(G)</td><td>1262.656</td><td>0.0032</td><td>2.5</td></tr>
<tr><td></td><td>1/4</td><td>2</td><td>20.77</td><td><0.1%</td><td>20.77</td><td>95.2</td><td>(K)L/S|E|V|N|P m/L/G F/R(G)</td><td>1262.656</td><td>15.9994</td><td>3.5</td></tr>
<tr><td>17</td><td>1/4</td><td>2</td><td>20.8</td><td><0.1%</td><td>14.88</td><td>96.6</td><td>(K)L/V|T|I/G/G H V/L/R(E)</td><td>1064.658</td><td>0.0028</td><td>2.7</td></tr>
<tr><td>18</td><td>4/4</td><td>2</td><td>27.03</td><td><0.1%</td><td>27.03</td><td>96.6</td><td>(K)L Y/G E|F|L|V|N/A/Q|G E/D/V/V/A/G I/R(T)</td><td>2050.06</td><td>0.0051</td><td>2.5</td></tr>
<tr><td>19</td><td>1/4</td><td>2</td><td>13.21</td><td><0.1%</td><td>13.21</td><td>76.2</td><td>(K)M V/E/P G H L D|Q\L L\H|P Q|F E/N|P S A Y\K(D)</td><td>2550.245</td><td>0.001</td><td>0.4</td></tr>
<tr><td>20</td><td>4/4</td><td>2</td><td>17.94</td><td><0.1%</td><td>17.94</td><td>85.9</td><td>(K)N/D|T|D|L|T/A/S/D/L/K(E)</td><td>1192.569</td><td>0.0029</td><td>2.5</td></tr>
<tr><td>21</td><td>3/4</td><td>2</td><td>19.03</td><td><0.1%</td><td>19.03</td><td>92.2</td><td>(R)N/N|G|A|Q|G/I|G/L/C/R(T)</td><td>1159.564</td><td>0.0029</td><td>2.5</td></tr>
<tr><td>22</td><td>4/4</td><td>2</td><td>24</td><td><0.1%</td><td>24</td><td>100</td><td>(K)Q L E\L|A|V|L|A|V|F|N/S/W/E/S/P R(A)</td><td>1959.033</td><td>0.0029</td><td>1.5</td></tr>
<tr><td>23</td><td>3/4</td><td>3</td><td>20.64</td><td><0.1%</td><td>20.64</td><td>96.1</td><td>(K)Q P L S P P A L\S\G D\L|G/T|F/M/A|W/V/D D/V/R(K)</td><td>2472.223</td><td>0.004</td><td>1.6</td></tr>
<tr><td></td><td>2/4</td><td>2</td><td>24.69</td><td><0.1%</td><td>20.89</td><td>89.1</td><td>(K)Q P/L|S|P P A L/S/G D/L/G T F/m A W/V/D/D/V/R(K)</td><td>2472.223</td><td>15.9942</td><td>-0.3</td></tr>
<tr><td>24</td><td>4/4</td><td>2</td><td>24.75</td><td><0.1%</td><td>24.75</td><td>90.3</td><td>(R)SGAA|M|S|M|PGMMDTV|L/N/L|GL/NDEV/A/A/GL/AAK(S)</td><td>2903.431</td><td>-0.0002</td><td>-0.1</td></tr>
<tr><td></td><td>3/4</td><td>2</td><td>24.32</td><td><0.1%</td><td>24.32</td><td>82.8</td><td>(R)SG/AAV|S/M|PGMmDTV|LN/L/GL/NDE/V/A/A/GL/AAK(S)</td><td>2903.431</td><td>15.9946</td><td>-0.1</td></tr>
<tr><td></td><td>1/4</td><td>2</td><td>24.92</td><td><0.1%</td><td>24.92</td><td>90.8</td><td>(R)SGAAV/S/m|PGM\M|D/TV|LN/L|GL/NDEV/A/A/GL/AAK(S)</td><td>2903.431</td><td>15.9952</td><td>0.1</td></tr>
<tr><td>25</td><td>4/4</td><td>2</td><td>21.38</td><td><0.1%</td><td>21.38</td><td>96.5</td><td>(R)T/E|H|M|F|F|A|S D|E/R(I)</td><td>1369.584</td><td>0.0034</td><td>2.5</td></tr>
<tr><td>26</td><td>4/4</td><td>2</td><td>21.4</td><td><0.1%</td><td>14.63</td><td>100</td><td>(R)T/P|E|D/L/D|A/M/K(D)</td><td>1019.471</td><td>0.0025</td><td>2.5</td></tr>
<tr><td></td><td>3/4</td><td>2</td><td>18.96</td><td><0.1%</td><td>18.96</td><td>78.8</td><td>(R)T/P|E/D|L/D|A/m/K(D)</td><td>1019.471</td><td>15.9981</td><td>3.1</td></tr>
<tr><td>27</td><td>1/4</td><td>2</td><td>14.3</td><td><0.1%</td><td>14.3</td><td>74.5</td><td>(K)V/G I C G E\H|G/G\E\P S S V|A|F|F/A|K(T)</td><td>1948.922</td><td>0.0036</td><td>1.8</td></tr>
<tr><td>28</td><td>2/4</td><td>2</td><td>17.69</td><td><0.1%</td><td>14.38</td><td>90.1</td><td>(K)V/G|T|M|I/E/I/P\R(A)</td><td>1015.561</td><td>0.0034</td><td>3.4</td></tr>
<tr><td></td><td>1/4</td><td>2</td><td>17.31</td><td><0.1%</td><td>17.31</td><td>82.2</td><td>(K)V/G|T|m|I E/I|P|R(A)</td><td>1015.561</td><td>15.9974</td><td>2.5</td></tr>
<tr><td>29</td><td>4/4</td><td>2</td><td>23.22</td><td><0.1%</td><td>23.22</td><td>100</td><td>(K)V/L|A|N|A|D|T|P D D/A L/T A R(N)</td><td>1542.776</td><td>0.0027</td><td>1.7</td></tr>
</table>

Evaluation of the 2D-electrophoretic procedure

Additional Figure A3. **Evaluation of 2D-electrophoretic reproducibility among protein samples from root system of maize plants exposed to the four nutritional treatments.** The graph shows the number of spots **(A)** and the sum of volume of all the spots **(B)** in the 2D-profile obtained from maize plants grown for 10 days without N sources and then exposed for the last 30 h to absence of N (c), to 10 mM NO_3^- (n), to 10 mM NH_4^+ (a) or to 5 mM NO_3^-+5 mM NH_4^+ (na). Data are expressed as average values ± SE (n=6). The upper letters indicate no differences among the four treatments according to Student's t-test (p<0.05).

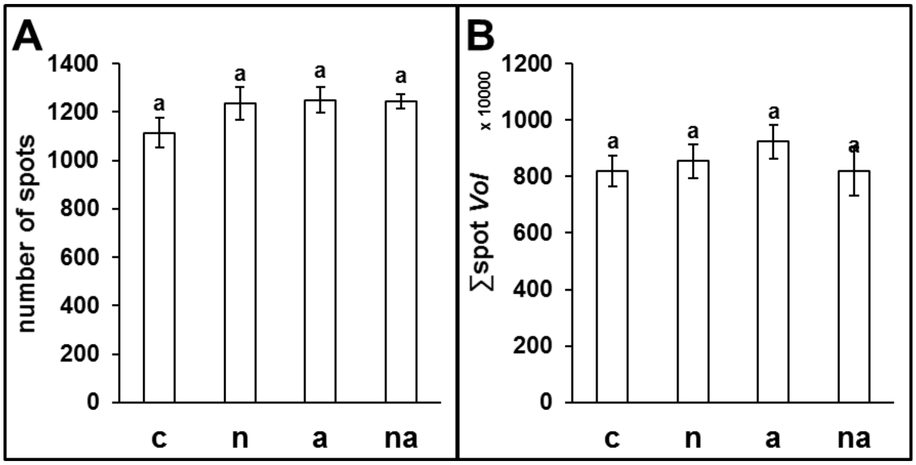

Spot characterization by LC-nESI-MS/MS

Supplementary data about spot identification by LC-nESI-MS/MS analysis. The results derive from four independent biological samples analysed in triplicate (n = 12). The data are the sums of all the replicates. In red are reported the sequenced peptides, among which the underlined ones are those that allowed discriminating among the different isoforms. The putative transit peptide is reported in green. In the table are reported the unique peptides with the best score assignment. **n**: spectrum number; **s**: specificity, those with the * are the discriminating peptides; **%f**: frequency of identification of the specific peptides among all the replicates (n=12); **z:** charge state; **Local FDR (%)**: Local False Discovery Rate (%); **SPI (%):** Score Peak Intensity (%); **Sequence map**: forward-slashes for locations of y-ions, backslashes for locations of b-ions, vertical lines for locations of both b- and y-ions. Spot numbers and acronyms refer to Fig. 3, Fig. 4 and Table 1 of the manuscript.

Additional Table A2

SPOT 1 – GS2a

>gi|121341|sp|P25462.1|GLNAC_MAIZE RecName: Full=Glutamine synthetase, chloroplastic; AltName: Full=GS2; AltName: Full=Glutamate--ammonia ligase; Flags: Precursor

MAQAVVPAMQCRVGVKAAAGRVWSAGRTRTGRGGASPGFKVMAVSTGSTGVVPRLEQLLNMDTTPYTDKVIAEYIWVGGSGIDIR
SKSRTISKPVEDPSELPKWNYDGSSTGQAPGEDSEVILYPQAIFKDPFRGGNNVLVICDTYTPQGEPLPTNKRHRAAQIFSDPKVGEQ
VPWFGIEQEYTLLQKDVNWPLGWPVGGFPGPQGPYYCAVGADKSFGRDISDAHYKACLYAGINISGTNGEVMPGQWEYQVGPSVG
IEAGDHIWISRYILERITEQAGVVLTLDPKPIQGDWNGAGCHTNYSTKTMREDGGFEEIKRAILNLSLRHDLHISAYGEGNERRLTGKH
ETASIGTFSWGVANRGCSIRVGRDTEAKGKGYLEDRRPASNMDPYIVTGLLAETTILWQPSLEAEALAAKKLALKV

94/423 AA: 22.2%
Total peptides (unique): 58 (9)
Isoform specific peptides: 9
Specificity reproducibility (%): 100%

<table>
<tr><th>n</th><th>s</th><th>%f</th><th>z</th><th>Score</th><th>Local FDR (%)</th><th>Database Fwd-Rev Score</th><th>SPI (%)</th><th>Sequence Map</th><th>MH^+ Matched (Da)</th><th>MH^+ Mass Shift (Da)</th><th>MH^+ Error (ppm)</th></tr>
<tr><td>1</td><td>*</td><td>83</td><td>2</td><td>19.48</td><td><0.1%</td><td>19.48</td><td>95.6</td><td>(R)A/A|Q|I|F|S/D|P K(V)</td><td>976.51</td><td>0.0043</td><td>4.4</td></tr>
<tr><td>2</td><td>*</td><td>58</td><td>2</td><td>17.24</td><td><0.1%</td><td>4.21</td><td>94.3</td><td>(R)A/I|L|N|L|S/L/R(H)</td><td>899.567</td><td>0.0044</td><td>4.9</td></tr>
<tr><td>3</td><td>*</td><td>33</td><td>2</td><td>19.17</td><td><0.1%</td><td>10.13</td><td>97.8</td><td>(R)D/I|S|D/A H|Y|K(A)</td><td>948.442</td><td>0.0042</td><td>4.4</td></tr>
<tr><td>4</td><td>*</td><td>8</td><td>2</td><td>15.86</td><td><0.1%</td><td>15.86</td><td>77.4</td><td>(R)E/D/G/G/F/E/E/I/K(R)</td><td>1023.463</td><td>0.0084</td><td>8.2</td></tr>
<tr><td>5</td><td>*</td><td>25</td><td>2</td><td>23.41</td><td><0.1%</td><td>17.39</td><td>91.8</td><td>(R)E/D/G/G/F/E/E/I/K/R(A)</td><td>1179.564</td><td>0.0047</td><td>4</td></tr>
<tr><td>6</td><td>*</td><td>25</td><td>3</td><td>17.88</td><td><0.1%</td><td>17.88</td><td>93.4</td><td>(R)H D L\H\I|S|A|Y|G/E|G N E R(R)</td><td>1597.735</td><td>0.0088</td><td>5.5</td></tr>
<tr><td>7</td><td>*</td><td>17</td><td>3</td><td>19.16</td><td><0.1%</td><td>19.16</td><td>97.9</td><td>(K)H E\T A S I\G\T|F|S/W/G/V/A N R(G)</td><td>1732.84</td><td>0.0078</td><td>4.5</td></tr>
<tr><td>8</td><td>*</td><td>50</td><td>2</td><td>24</td><td><0.1%</td><td>24</td><td>100</td><td>(R)L E|Q|L|L|N|M|D/T/T|P Y/T/D K(V)</td><td>1781.863</td><td>0.0077</td><td>4.3</td></tr>
<tr><td>9</td><td>*</td><td>25</td><td>3</td><td>20.77</td><td><0.1%</td><td>15.97</td><td>97.3</td><td>(R)T/I|S K/P V/E/D|P S E/L/P K(W)</td><td>1539.826</td><td>0.0087</td><td>5.6</td></tr>
</table>

Additional Table A3

SPOT 2 – GS2b

>gi|121341|sp|P25462.1|GLNAC_MAIZE RecName: Full=Glutamine synthetase, chloroplastic; AltName: Full=GS2; AltName: Full=Glutamate--ammonia ligase; Flags: Precursor

MAQAVVPAMQCRVGVKAAAGRVWSAGRTRTGRGGASPGFKVMAVSTGSTGVVPRLEQLLNMDTTPYTDKVIAEYIWVGGSGIDIR
SKSRTISKPVEDPSELPKWNYDGSSTGQAPGEDSEVILYPQAIFKDPFRGGNNVLVICDTYTPQGEPLPTNKRHRAAQIFSDPKVGEQ
VPWFGIEQEYTLLQKDVNWPLGWPVGGFPGPQGPYYCAVGADKSFGRDISDAHYKACLYAGINISGTNGEVMPGQWEYQVGPSVG
IEAGDHIWISRYILERITEQAGVVLTLDPKPIQGDWNGAGCHTNYSTKTMREDGGFEEIKRAILNLSLRHDLHISAYGEGNERRLTGKH
ETASIGTFSWGVANRGCSIRVGRDTEAKGKGYLEDRRPASNMDPYIVTGLLAETTILWQPSLEAEALAAKKLALKV

*: single mismatch (G → A) from >unnamed protein product (CAY33789.1). This mutation does not modify the p*I* of the protein.

115/423 AA: 27.2% coverage
Total peptides (unique): 95 (10)
Isoform specific peptides: 10
Specificity reproducibility (%): 100%

<table>
<tr><th>n</th><th>s</th><th>%f</th><th>z</th><th>Score</th><th>Local FDR (%)</th><th>Database Fwd-Rev Score</th><th>SPI (%)</th><th>Sequence Map</th><th>MH^+ Matched (Da)</th><th>MH^+ Mass Shift (Da)</th><th>MH^+ Error (ppm)</th></tr>
<tr><td>1</td><td>*</td><td>100</td><td>2</td><td>20.06</td><td><0.1%</td><td>20.06</td><td>97</td><td>(R)A A|Q|I|F|S/D|P/K(V)</td><td>976.51</td><td>0.0023</td><td>2.4</td></tr>
<tr><td>2</td><td>*</td><td>92</td><td>2</td><td>18.37</td><td><0.1%</td><td>3.23</td><td>97.5</td><td>(R)A/I|L|N|L|S/L/R(H)</td><td>899.567</td><td>0.0018</td><td>2.1</td></tr>
<tr><td>3</td><td>*</td><td>42</td><td>2</td><td>19.09</td><td><0.1%</td><td>11.44</td><td>95.2</td><td>(R)D/I|S|D/A/H|Y/K(A)</td><td>948.442</td><td>0.0014</td><td>1.5</td></tr>
<tr><td>4</td><td>*</td><td>92</td><td>2</td><td>18.24</td><td><0.1%</td><td>18.24</td><td>87.8</td><td>(R)E/D/G/G/F/E/E/I/K(R)</td><td>1023.463</td><td>0.0044</td><td>4.3</td></tr>
<tr><td>5</td><td>*</td><td>67</td><td>2</td><td>20.35</td><td><0.1%</td><td>20.35</td><td>83.7</td><td>(R)E/D|G/G/F/E/E|I/K/R(A)</td><td>1179.564</td><td>0.0057</td><td>4.8</td></tr>
<tr><td>6</td><td>*</td><td>33</td><td>3</td><td>15.03</td><td><0.1%</td><td>15.03</td><td>85.2</td><td>(K)H E T A S\I\G T\F/S|W/G/V/A N R(G)</td><td>1732.84</td><td>0.0018</td><td>1</td></tr>
<tr><td>7</td><td>*</td><td>100</td><td>2</td><td>27.25</td><td><0.1%</td><td>27.25</td><td>100</td><td>(R)L E|Q|L|L|N/m/D/T/T|P Y/T/D/K(V)</td><td>1781.863</td><td>16.0087</td><td>7.7</td></tr>
<tr><td>8</td><td>*</td><td>92</td><td>2</td><td>18.58</td><td><0.1%</td><td>18.58</td><td>98.4</td><td>(R)T I/S/K|P V E D|P S E|L|P/K(W)</td><td>1539.826</td><td>0.0081</td><td>5.2</td></tr>
<tr><td>9</td><td>*</td><td>33</td><td>3</td><td>19.94</td><td><0.1%</td><td>19.94</td><td>85</td><td>(K)V A E Q\V|P W|F\G\I/E/Q/E/Y/T/L/L/Q K(D)</td><td>2278.175</td><td>0.0029</td><td>1.3</td></tr>
<tr><td>10</td><td>*</td><td>25</td><td>2</td><td>15.66</td><td><0.1%</td><td>15.66</td><td>83.3</td><td>(K)V I A E|Y|I|W V|G/G/S/G I D/I R(S)</td><td>1747.938</td><td>0.0056</td><td>3.2</td></tr>
</table>

Additional Table A4

SPOT 3 – GS2c

>gi|121341|sp|P25462.1|GLNAC_MAIZE RecName: Full=Glutamine synthetase, chloroplastic; AltName: Full=GS2; AltName: Full=Glutamate--ammonia ligase; Flags: Precursor

MAQAVVPAMQCRVGVKAAAGRVWSAGRTRTGRGGASPGFKVMAVSTGSTGVVPRLEQLLNMDTTPYTDKVIAEYIWVGGSGIDIR
SKSRTISKPVEDPSELPKWNYDGSSTGQAPGEDSEVILYPQAIFKDPFRGGNNVLVICDTYTPQGEPLPTNKRHRAAQIFSDPKVGEQ
VPWFGIEQEYTLLQKDVNWPLGWPVGGFPGPQGPYYCAVGADKSFGRDISDAHYKACLYAGINISGTNGEVMPGQWEYQVGPSVG
IEAGDHIWISRYILERITEQAGVVLTLDPKPIQGDWNGAGCHTNYSTKTMREDGGFEEIKRAILNLSLRHDLHISAYGEGNERRLTGKH
ETASIGTFSWGVANRGCSIRVGRDTEAKGKGYLEDRRPASNMDPYIVTGLLAETTILWQPSLEAEALAAKKLALKV

46/423 AA: 10.9% coverage
Total peptides (unique): 46 (4)
Isoform specific peptides: 4
Specificity reproducibility (%): 100%

n	s	%f	z	Score	Local FDR (%)	Database Fwd-Rev Score	SPI (%)	Sequence Map	MH^+ Matched (Da)	MH^+ Mass Shift (Da)	MH^+ Error (ppm)
1	*	100	2	20.89	<0.1%	20.89	98.5	(R)A/A|Q|I|F|S/D|P/K(V)	976.51	0.0003	0.3
2	*	42	2	18.94	<0.1%	10.03	91.3	(R)D/I|S|D/A/H|Y/K(A)	948.442	0.0024	2.5
3	*	100	2	24.14	<0.1%	18.84	98.4	(R)L E|Q|L|L|N|M|D/T/T|P Y/T/D/K(V)	1781.863	0.0043	2.4
4	*	25	2	16.66	<0.1%	16.66	87.3	(R)T I/S|K|P V/E D|P S E|L|P K(W)	1539.826	-0.0013	-0.9

Additional Table A5

SPOT 4 – GS1-3/4

Co-migration of GS1-3 + GS1-4

>gi|585204|sp|P38562.1|GLNA4_MAIZE RecName: Full=Glutamine synthetase root isozyme 4; AltName: Full=GS107; AltName: Full=Glutamate--ammonia ligase

MACLTDLVNLNLSDTTEKIIAEYIWIGGSGMDLRSKARTLPGPVTDPSKLPKWNYDGSSTGQAPGEDSEVILYPQAIFKDPFRRGNNIL
VMCDCYTPAGEPIPTNKRYSAAKIFSSPEVAAEEPWYGIEQEYTLLQKDTNWPLGWPIGGFPGPQGPYYCGIGAEKSFGRDIVDAHY
KACLYAGINISGINGEVMPGQWEFQVGPSVGISSGDQVWVARYILERITEIAGVVVTFDPKPIPGDWNGAGAHTNYSTESMRKEGGY
EVIKAAIEKLKLRHKEHIAAYGEGNERRLTGRHETADINTFSWGVANRGASVAVGQTEQNGKGYFEDRRPASNMDPYVVTSMIAETTI
VWKP

* in GS1-3: TLSGPVTDPSK, in GS1-4: TLPGPVTDPSK. Both sequences were sequenced.
** in GS1-3: RPASNMDPYVVTSMIAETTIIWKP, in GS1-4: RPASNMDPYVVTSMIAETTIVWKP. Both sequences were sequenced.

GS1-3: 166/356 AA: 46.6%
GS1-4: 180/355 AA: 50.7%
GS1-3 Total peptides (unique): 145 (10)
GS1-4 Total peptides (unique): 178 (12)
Isoform specific peptides: 6 common + 3 for GS1-3 + 3 for GS1-4
Specificity reproducibility (%): 100%

n	s	%f	z	Score	Local FDR (%)	Database Fwd-Rev Score	SPI (%)	Sequence Map	MH^+ Matched (Da)	MH^+ Error (ppm)
1	*	75	2	20.65	<0.1%	11.06	97.6	(R)D/I|V|D/A/H|Y/K(A)	960.479	1.5
2	*	17	3	20.36	<0.1%	20.36	83.9	(K)D T N W|P\L\G|W|P I/G G/F/P G/P Q/G P Y Y C G\I/G A E K(S)	3034.419	3.8
3	*	100	2	19.5	<0.1%	14.56	89.2	(K)E/G/G/Y/E/V/I/K(A)	894.457	1.1
4			3	21.65	<0.1%	21.65	98.1	(K)E H\I\A A|Y|G|E/G/N/E/R(R)	1345.613	2
5	*	25	2	24.54	<0.1%	24.54	96.2	(R)H/E|T|A|D|I|N|T|F/S/W/G V/A N R(G)	1817.857	4.2
6			2	17.11	<0.1%	17.11	79.8	(R)H/K|E|H|I|A|A/Y|G E G N E/R(R)	1610.767	5.4
7	*	100	3	25.71	<0.1%	25.71	97.4	(K)I F S S P E V\A\A E\E|P W/Y/G I/E/Q/E/Y/T/L/L/Q/K(D)	2927.435	5.6
8	*	100	2	23.19	<0.1%	23.19	94.9	(K)I I|A E|Y|I|W/I|G/G/S G/M/D/L/R(S)	1793.926	2
9	*3	100	3	18.48	<0.1%	18.48	91.6	(R)R P A S N M D\P\Y\V\V\T S m I/A/E\T T I|I|W|K/P(-)	2720.379	5.8
10	*4	50	3	14.46	<0.1%	14.46	84.5	(R)R P A S N m D P Y V\V\T S m I/A\E\T T I|V|W/K/P(-)	2706.363	3.6
11	*4	67	2	23.2	<0.1%	15.29	99.7	(R)T/L|P|G/P/V T/D/P S/K(L)	1111.599	3.2
12	*4	42	2	22.41	<0.1%	15.66	97.1	(R)T/L|P/G/P V/T/D/P S K/L|P K(W)	1449.831	3.6
13	*3	92	2	21.17	<0.1%	12.47	100	(R)T/L|S|G|P V T/D|P S/K(L)	1101.579	3.9
14	*3	50	2	21.41	<0.1%	16.34	91.9	(R)T L|S/G/P V/T/D|P S K/L|P K(W)	1439.81	3
15			2	24.52	<0.1%	24.52	93.5	(K)W N/Y D/G S S T/G Q|A|P G E D/S E/V/I/L|Y|P Q/A/I/F K(D)	2972.395	3.2

Additional Table A6

SPOT 5 – GS1-5

>gi|1169926|sp|P38563.2|GLNA5_MAIZE RecName: Full=Glutamine synthetase root isozyme 5; AltName: Full=GS117; AltName: Full=Glutamate--ammonia ligase

MASLTDLVNLDLSDCTDKIIAEYIWVGGSGIDLRSKARTVKGPITDPSQLPKWNYDGSSTGQAPGEDSEVILYPQAIFKDPFRKGNNIL
VMCDCYTPQGEPIPSNKRYKAATVFSHPDVAAEVPWYGIEQEYTLLQKDLSWPLGWPVGGYPGPQGPYYCAAGADKAFGRDVVDA
HYKACLYAGINISGINGEVMPGQWEFQVGPSVGISAGDEIWVARYILERITEMAGIVLSLDPKPIKGDWNGAGAHTNYSTKSMREAGG
YEVIKEAIEKLGRRHREHIAAYGEGNERRLTGRHETADINTFKWGVANRGASIRVGRDTEKEGKGYFEDRRPASNMDPYVVTGMIAD
TTILWKGN

184/357 AA: 51.5% coverage
Total peptides (unique): 301 (14)
Isoform specific peptides: 6
Specificity reproducibility (%): 100%

<table>
<tr><th>n</th><th>s</th><th>%f</th><th>z</th><th>Score</th><th>Local FDR (%)</th><th>Database Fwd-Rev Score</th><th>SPI (%)</th><th>Sequence Map</th><th>MH+ Matched (Da)</th><th>MH+ Error (ppm)</th></tr>
<tr><td>1</td><td>*</td><td>100</td><td>3</td><td>24.7</td><td><0.1%</td><td>17.33</td><td>100</td><td>(K)AATVFSHPDVAA\E|M|PW/Y/GI/E/QE/Y/T/L/LQ/K(D)</td><td>3162.578</td><td>6.3</td></tr>
<tr><td>2</td><td></td><td></td><td>2</td><td>21.17</td><td><0.1%</td><td>13.49</td><td>97.5</td><td>(R)D/V|V|D/A/H|Y/K(A)</td><td>946.463</td><td>0.9</td></tr>
<tr><td>3</td><td></td><td></td><td>2</td><td>18.47</td><td><0.1%</td><td>11.95</td><td>88.7</td><td>(R)E A|G/G/Y/E/V/I/K(A)</td><td>965.494</td><td>1.1</td></tr>
<tr><td>4</td><td>*</td><td>8</td><td>3</td><td>14.67</td><td><0.1%</td><td>9.3</td><td>79.2</td><td>(R)E A/G/G/Y/E/V I/K E A I E/K(L)</td><td>1535.795</td><td>3.2</td></tr>
<tr><td>5</td><td></td><td></td><td>3</td><td>20.92</td><td><0.1%</td><td>20.92</td><td>98.5</td><td>(K)E H\I\A A|Y|G|E/G/N E/R(R)</td><td>1345.613</td><td>1.7</td></tr>
<tr><td>6</td><td></td><td></td><td>3</td><td>12.05</td><td><0.1%</td><td>12.05</td><td>70.6</td><td>(K)E H|I/A/A Y/G E/G N E R|R(L)</td><td>1501.714</td><td>1.1</td></tr>
<tr><td>7</td><td></td><td></td><td>2</td><td>24.05</td><td><0.1%</td><td>24.05</td><td>89.4</td><td>(K)G D/W/N/G/A|G A/H|T N/Y/S|T/K(S)</td><td>1578.693</td><td>9.2</td></tr>
<tr><td>8</td><td>*</td><td>100</td><td>2</td><td>19.08</td><td><0.1%</td><td>19.08</td><td>98.4</td><td>(K)G/P|I|T|D|P S Q/L|P K(W)</td><td>1152.626</td><td>3.1</td></tr>
<tr><td>9</td><td></td><td></td><td>2</td><td>20.08</td><td><0.1%</td><td>20.08</td><td>97.7</td><td>(R)H|E|T|A D|I|N|T|F/K(W)</td><td>1175.569</td><td>1.7</td></tr>
<tr><td>10</td><td>*</td><td>100</td><td>4</td><td>19.75</td><td><0.1%</td><td>19.75</td><td>96.5</td><td>(R)H R/E H\I\A\A Y|G|E|G/N/E/R(R)</td><td>1638.773</td><td>-1.1</td></tr>
<tr><td>11</td><td>*</td><td>100</td><td>2</td><td>23.89</td><td><0.1%</td><td>23.89</td><td>98</td><td>(R)I I|A/E|Y|I|W\V|G|G/S/G I/D/L R(S)</td><td>1761.953</td><td>2.8</td></tr>
<tr><td>12</td><td></td><td></td><td>3</td><td>25.32</td><td><0.1%</td><td>21.07</td><td>97</td><td>(R)I/T|E/M/A/G I/V/L/S/L/D/P K/P I/K(G)</td><td>1825.05</td><td>2.1</td></tr>
<tr><td>13</td><td>*</td><td>100</td><td>3</td><td>24.5</td><td><0.1%</td><td>24.5</td><td>100</td><td>(R)R P A S N M D\P Y\V\V|T|G/m|I|A/D T T/I/L/W/K(G)</td><td>2579.3</td><td>5.5</td></tr>
<tr><td>14</td><td></td><td></td><td>3</td><td>20.38</td><td><0.1%</td><td>20.38</td><td>98.3</td><td>(K)WNYDGSSTGQ|A|PGEDSE|M|L/Y/PQAI/FK(D)</td><td>2972.395</td><td>4.4</td></tr>
</table>

Additional Table A7

SPOT 6 – GS1-1

>gi|585201|sp|P38559.1|GLNA1_MAIZE RecName: Full=Glutamine synthetase root isozyme 1; AltName: Full=GS122; AltName: Full=Glutamate--ammonia ligase

MASLTDLVNLDLSDCTDRIIAEYIWIGGTGIDLRSKARTVKGPITDPI*QLPKWNYDGSSTGQAPGEDSEVILYPQAIFKDPFRKGNHILV
MCDCYTPQGEPIPTNKRYSAAKVFSHPDVAAEVPWYGIEQEYTLLQKDVSWPLGWPVGGYPGPQGPYYCAAGADKAFGRDVVDA
HYKACLYAGINISGINGEVMPGQWEFQVGPSVGISAGDEIWVARYILERITEMAGIVLSLDPKPIKGDWNGAGAHTNYSTKSMREAGG
YEVIKAAIDKLGKRHKEHIAAYGEGNERRLTGRHETADINTFKWGVANRGASIRVGRDTEREGKGYFEDRRPASNMDPYVVTGMIAE
TTILWNGN

*: with the sequence conflict (I → S) reported in SwissProt Reviewed Database. This mutation does not modify the p*I* of the protein.

186/357 AA: 52.1% coverage
Total peptides (unique): 255 (17)
Isoform specific peptides: 3
Specificity reproducibility (%): 100%

<table>
<tr><th>n</th><th>s</th><th>%f</th><th>z</th><th>Score</th><th>Local FDR (%)</th><th>Database Fwd-Rev Score</th><th>SPI (%)</th><th>Sequence Map</th><th>MH+ Matched (Da)</th><th>MH+ Mass Shift (Da)</th><th>MH+ Error (ppm)</th></tr>
<tr><td>1</td><td></td><td></td><td>2</td><td>21.42</td><td><0.1%</td><td>13.78</td><td>97.5</td><td>(R)D/V|V|D/A/H|Y/K(A)</td><td>946.463</td><td>0.0013</td><td>1.3</td></tr>
<tr><td>2</td><td></td><td></td><td>2</td><td>18.24</td><td><0.1%</td><td>9.68</td><td>88.8</td><td>(R)E A|G/G/Y/E/V/I/K(A)</td><td>965.494</td><td>0.0019</td><td>2</td></tr>
<tr><td>3</td><td></td><td></td><td>3</td><td>21</td><td><0.1%</td><td>21</td><td>100</td><td>(K)E H\I\A A|Y|G|E/G/N/E/R(R)</td><td>1345.613</td><td>0.0041</td><td>3.1</td></tr>
<tr><td>4</td><td></td><td></td><td>3</td><td>10.39</td><td><0.1%</td><td>10.39</td><td>77.4</td><td>(K)E H/I|A/A Y/G E/G N E R/R(L)</td><td>1501.714</td><td>0.0035</td><td>2.3</td></tr>
<tr><td>5</td><td></td><td></td><td>2</td><td>24.66</td><td><0.1%</td><td>24.66</td><td>91.3</td><td>(K)G D/W/N/G/A|G/A/H T\N/Y|S/T/K(S)</td><td>1578.693</td><td>0.0016</td><td>1</td></tr>
<tr><td>6</td><td></td><td></td><td>2</td><td>18.05</td><td><0.1%</td><td>18.05</td><td>98.1</td><td>(K)G/P|I|T|D|P I Q|L|P K(W)</td><td>1178.678</td><td>0.0022</td><td>1.8</td></tr>
<tr><td>7</td><td></td><td></td><td>2</td><td>18.37</td><td><0.1%</td><td>18.37</td><td>99</td><td>(K)G/P|I|T|D|P S Q/L|P K(W)</td><td>1152.626</td><td>0.001</td><td>0.9</td></tr>
<tr><td>8</td><td></td><td></td><td>2</td><td>20.1</td><td><0.1%</td><td>20.1</td><td>93.8</td><td>(R)H|E|T|A/D|I|N|T|F|K(W)</td><td>1175.569</td><td>0.0004</td><td>0.3</td></tr>
<tr><td>9</td><td></td><td></td><td>3</td><td>17.88</td><td><0.1%</td><td>17.88</td><td>90.7</td><td>(R)H K/E H I|A|A|Y|G\E|G N E/R(R)</td><td>1610.767</td><td>0.0053</td><td>3.3</td></tr>
<tr><td>10</td><td></td><td></td><td>4</td><td>15.79</td><td><0.1%</td><td>15.79</td><td>86.2</td><td>(R)H/K/E H|I|A/A\Y/G\E/G N E R R(L)</td><td>1766.868</td><td>0.0009</td><td>0.5</td></tr>
<tr><td>11</td><td>*</td><td>67</td><td>2</td><td>21.7</td><td><0.1%</td><td>21.7</td><td>95.2</td><td>(R)I I/A\E|Y|I|W/I|G|G T G I/D/L/R(S)</td><td>1789.985</td><td>0.003</td><td>1.7</td></tr>
<tr><td>12</td><td></td><td></td><td>3</td><td>26.59</td><td><0.1%</td><td>22.59</td><td>97.6</td><td>(R)I/T|E|M/A/G I/V/L/S L/D/P K/P I K(G)</td><td>1825.05</td><td>0.0053</td><td>2.9</td></tr>
<tr><td>13</td><td>*</td><td>33</td><td>3</td><td>16.51</td><td><0.1%</td><td>16.51</td><td>92</td><td>(R)R P A S N m D P Y V\V\T\G m\I\A|E\T T I/L|W/N G/N(-)</td><td>2750.328</td><td>31.9902</td><td>0.1</td></tr>
<tr><td>14</td><td></td><td></td><td>2</td><td>17.68</td><td><0.1%</td><td>17.68</td><td>88.1</td><td>(R)T V/K|G|P I|T\D|P S Q/L|P K(W)</td><td>1480.837</td><td>0.0036</td><td>2.4</td></tr>
<tr><td>15</td><td>*</td><td>100</td><td>3</td><td>27.52</td><td><0.1%</td><td>27.52</td><td>95.8</td><td>(K)V F S H P D V A A E\V|P W/Y|G|I/E/Q/E/Y/T/L/L/Q/K(D)</td><td>2919.456</td><td>0.0137</td><td>4.7</td></tr>
<tr><td>16</td><td></td><td></td><td>2</td><td>23.53</td><td><0.1%</td><td>23.53</td><td>93.6</td><td>(K)W N Y D/G S S T/G Q|A|P G E D/S E/V/I/L|Y|P Q/A/I/F K(D)</td><td>2972.395</td><td>0.0113</td><td>3.8</td></tr>
<tr><td>17</td><td></td><td></td><td>4</td><td>14.64</td><td><0.1%</td><td>14.64</td><td>74.2</td><td>(K)W N\Y D G S S T G Q/A|P G E/D S E/V/I L Y/P Q A I F K D/P F R K(G)</td><td>3615.739</td><td>0.0171</td><td>4.7</td></tr>
</table>

Evaluation of amino acid levels by LC-ESI-MS

Maize plants were grown for 10 days without N sources and then exposed for the last 30 h to absence of N (c), to 10 mM NO_3^- (n), to 10 mM NH_4^+ (a) or to 5 mM NO_3^-+5 mM NH_4^+ (na). **TOT**: calculated sum of total amino acids. The table reports the amino acid concentrations (µM) as average values ± SE and the proportion of total amino acids (%). The analyses were conducted on four roots and leaves and three xylem sap biological samples analyzed in duplicate (n=8, n=6).The asterisk indicates significant differences with respect to (c) plants (p column, Student's t-test with: ** p≤0.01, * p≤0.05).

Additional Table A8. Levels of amino acids in roots

	c plants			n plants				a plants				na plants			
	µM	SE	%	µM	SE	%	p	µM	SE	%	p	µM	SE	%	p
GLY	137.5	35.6	3.78	74.8	12.9	1.04		111.0	11.6	0.79		82.9	5.6	0.65	
ALA	1072.2	179.4	29.47	2827.4	354.4	39.37	**	3948.1	559.0	28.07	**	5077.7	727.3	39.86	**
SER	418.6	93.1	11.50	351.2	36.6	4.89		239.6	33.5	1.70		301.1	26.9	2.36	
PRO	9.3	1.7	0.25	6.3	0.6	0.09		9.6	1.5	0.07		6.2	0.7	0.05	
VAL	95.0	4.1	2.61	91.6	9.0	1.28		154.2	9.0	1.10	**	146.8	6.0	1.15	**
THR	93.0	10.6	2.56	78.0	5.6	1.09		86.7	9.2	0.62		93.2	3.9	0.73	
ILE	89.3	4.3	2.45	34.5	2.6	0.48	**	54.0	4.1	0.38	**	65.9	10.2	0.52	*
LEU	170.7	8.3	4.69	38.6	2.2	0.54	**	78.1	8.0	0.56	**	94.0	21.8	0.74	**
ASN	230.5	75.7	6.34	298.2	51.8	4.15		4404.7	634.2	31.32	**	2093.3	323.0	16.43	**
ASP	210.2	26.6	5.78	353.5	29.0	4.92	**	292.5	46.7	2.08		332.0	30.5	2.61	**
GLN	301.2	41.6	8.28	945.2	62.2	13.16	**	3771.8	618.1	26.82	**	3024.3	270.4	23.74	**
LYS	43.7	2.0	1.20	22.4	1.3	0.31	**	38.1	2.3	0.27		37.7	3.0	0.30	
GLU	428.0	94.5	11.76	1856.0	215.1	25.85	**	602.7	156.6	4.28		1098.4	129.0	8.62	**
MET	10.4	0.7	0.29	6.4	0.5	0.09	**	8.0	0.7	0.06	*	7.5	0.9	0.06	*
HIS	135.7	26.9	3.73	42.4	6.0	0.59	**	64.2	9.0	0.46	*	78.2	22.1	0.61	
PHE	36.7	1.3	1.01	29.8	1.7	0.42	**	37.9	1.7	0.27		38.8	2.8	0.30	
ARG	44.9	2.6	1.23	27.7	2.0	0.39	**	44.1	2.9	0.31		44.4	4.4	0.35	
TYR	47.4	4.3	1.30	50.2	4.7	0.70		67.7	3.2	0.48	**	68.5	7.3	0.54	*
TRP	59.7	2.0	1.64	39.7	2.8	0.55	**	46.8	1.4	0.33	**	45.5	3.8	0.36	**
CYS	4.8	0.7	0.13	7.5	2.1	0.10		5.4	1.8	0.04		3.6	0.2	0.03	
TOT	3638.8	376.2	100.0	7181.4	570.6	100.0	**	14065.2	2021.8	100.0	**	12740.0	521.5	100.0	**
Δ%c						+97.4				+286.53				+250.1	

Additional Table A9. Levels of amino acids in xylem sap

	c plants			n plants				a plants				na plants			
	µM	SE	%	µM	SE	%	p	µM	SE	%	p	µM	SE	%	p
GLY	**13.5**	1.8	**0.58**	**13.5**	0.6	**0.15**		**9.5**	0.5	**0.1**	*	**7.5**	0.3	**0.05**	**
ALA	**615.0**	98.6	**26.60**	**2987.7**	131.3	**32.35**	**	**3606.2**	262.4	**29.3**	**	**6831.2**	269.9	**43.87**	**
SER	**303.1**	101.3	**13.11**	**269.4**	19.6	**2.92**		**73.4**	3.2	**0.6**	*	**81.8**	3.2	**0.53**	
PRO	**19.8**	3.8	**0.85**	**18.9**	1.4	**0.20**		**31.0**	2.6	**0.3**	*	**22.8**	1.4	**0.15**	
VAL	**120.3**	19.5	**5.21**	**148.1**	10.2	**1.60**		**238.8**	21.4	**1.9**	**	**237.4**	15.1	**1.52**	**
THR	**112.7**	26.0	**4.88**	**207.1**	10.3	**2.24**	**	**102.3**	3.3	**0.8**		**98.8**	1.2	**0.63**	
ILE	**92.8**	17.7	**4.01**	**65.2**	4.9	**0.71**		**131.5**	14.8	**1.1**		**102.9**	6.6	**0.66**	
LEU	**91.7**	27.5	**3.97**	**52.9**	7.4	**0.57**		**125.9**	16.7	**1.0**		**75.0**	5.6	**0.48**	
ASN	**84.0**	32.4	**3.64**	**1042.6**	45.3	**11.29**	**	**591.4**	23.3	**4.8**	**	**779.6**	134.3	**5.01**	**
ASP	**28.9**	3.3	**1.25**	**32.2**	5.5	**0.35**		**28.3**	3.3	**0.2**		**17.4**	4.4	**0.11**	*
GLN	**407.6**	100.6	**17.63**	**3778.3**	256.5	**40.91**	**	**6552.6**	281.7	**53.2**	**	**6512.3**	197.8	**41.82**	**
LYS	**166.9**	27.8	**7.22**	**136.7**	7.5	**1.48**		**238.9**	19.3	**1.9**	*	**206.3**	12.9	**1.32**	
GLU	**30.2**	4.4	**1.31**	**71.2**	6.4	**0.77**	**	**87.1**	4.1	**0.7**	**	**70.8**	10.9	**0.45**	**
MET	**8.2**	2.1	**0.35**	**49.3**	2.8	**0.53**	**	**36.9**	4.1	**0.3**	**	**32.5**	1.0	**0.21**	**
HIS	**86.3**	12.4	**3.73**	**134.0**	10.8	**1.45**	**	**88.4**	11.9	**0.7**		**125.5**	7.7	**0.81**	*
PHE	**25.6**	5.9	**1.11**	**44.7**	5.2	**0.48**	*	**116.0**	11.4	**0.9**	**	**72.2**	5.5	**0.46**	**
ARG	**50.2**	11.7	**2.17**	**133.7**	5.6	**1.45**	**	**152.8**	13.6	**1.2**	**	**218.8**	12.5	**1.41**	**
TYR	**18.1**	4.8	**0.78**	**20.5**	0.8	**0.22**		**86.2**	12.6	**0.7**	**	**61.5**	4.2	**0.39**	**
TRP	**17.2**	1.5	**0.74**	**12.4**	0.5	**0.13**	**	**17.7**	0.6	**0.1**		**12.3**	0.7	**0.08**	**
CYS	**19.3**	2.5	**0.84**	**16.2**	0.5	**0.18**		**8.5**	0.5	**0.1**	**	**4.9**	0.5	**0.03**	**
TOT	**2311.6**	493.5	**100.0**	**9234.7**	405.7	**100.0**	**	**12323.3**	625.8	**100.0**	**	**15571.4**	432.7	**100.0**	**
Δ%c						**+299.5**				**+433.1**				**+573.6**	

Additional Table A10. Levels of amino acids in leaves

	c plants			n plants				a plants				na plants			
	µM	SE	%	µM	SE	%	p	µM	SE	%	p	µM	SE	%	p
GLY	**1896.3**	694.1	**33.62**	**870.9**	32.4	**10.71**		**2727.3**	590.6	**14.18**		**2555.1**	311.6	**12.35**	
ALA	**1913.0**	161.9	**33.92**	**3461.6**	172.0	**42.55**	**	**9786.3**	2240.8	**50.88**	**	**11431.6**	2011.3	**55.24**	**
SER	**230.7**	17.6	**4.09**	**362.5**	16.2	**4.46**	**	**606.7**	97.8	**3.15**	**	**829.6**	41.7	**4.01**	**
PRO	**3.2**	1.3	**0.06**	**4.9**	1.5	**0.06**		**9.5**	2.1	**0.05**	*	**12.1**	2.9	**0.06**	**
VAL	**325.6**	18.3	**5.77**	**330.1**	5.2	**4.06**		**457.7**	6.2	**2.38**	**	**433.1**	18.4	**2.09**	**
THR	**109.8**	4.9	**1.95**	**97.8**	2.7	**1.20**	*	**165.6**	7.3	**0.86**	**	**157.7**	7.0	**0.76**	**
ILE	**9.9**	0.3	**0.17**	**9.2**	0.3	**0.11**		**37.1**	7.3	**0.19**	**	**36.3**	1.7	**0.18**	**
LEU	**7.5**	1.3	**0.13**	**3.6**	0.6	**0.04**	**	**23.0**	6.0	**0.12**	*	**21.2**	0.9	**0.10**	**
ASN	**42.9**	1.7	**0.76**	**41.1**	0.9	**0.51**		**399.9**	110.8	**2.08**	**	**275.9**	78.6	**1.33**	**
ASP	**94.4**	2.4	**1.67**	**266.6**	27.2	**3.28**	**	**266.3**	15.0	**1.38**	**	**262.5**	12.3	**1.27**	**
GLN	**60.7**	3.2	**1.08**	**194.1**	5.9	**2.39**	**	**867.2**	212.0	**4.51**	**	**851.4**	104.9	**4.11**	**
LYS	**21.8**	1.5	**0.39**	**15.8**	2.5	**0.19**	*	**36.8**	8.8	**0.19**		**32.4**	7.3	**0.16**	
GLU	**735.8**	170.2	**13.04**	**2265.4**	670.0	**27.85**	*	**3519.4**	1056.8	**18.30**	*	**3520.0**	1012.0	**17.01**	**
MET	**6.0**	0.8	**0.11**	**6.1**	0.4	**0.07**		**11.4**	1.0	**0.06**	**	**12.7**	0.5	**0.06**	**
HIS	**114.5**	27.2	**2.03**	**138.2**	55.6	**1.70**		**161.4**	52.5	**0.84**		**107.7**	37.5	**0.52**	
PHE	**8.4**	0.9	**0.15**	**12.8**	0.3	**0.16**	**	**40.3**	8.4	**0.21**	**	**38.2**	1.9	**0.18**	**
ARG	**1.3**	0.1	**0.02**	**1.2**	0.1	**0.01**		**1.5**	0.2	**0.01**		**3.7**	1.8	**0.02**	
TYR	**16.9**	0.9	**0.30**	**25.4**	0.4	**0.31**	**	**64.0**	13.2	**0.33**	**	**67.5**	2.1	**0.33**	**
TRP	**37.2**	1.9	**0.66**	**24.0**	2.6	**0.29**	**	**46.7**	4.6	**0.24**		**42.8**	3.3	**0.21**	
CYS	**4.8**	1.1	**0.09**	**3.7**	0.3	**0.05**		**4.5**	1.0	**0.02**		**4.1**	0.5	**0.02**	
TOT	**5640.6**	1008.4	**100.0**	**8135.0**	729.8	**100.0**	*	**19232.6**	4312.2	**100.0**	**	**20695.8**	3458.7	**100.0**	**
Δ%c						**+44.2**				**+240.97**				**+266.9**	

Additional Table A11. List of experimental parameters used for amino acid quantitation. The standards were prepared to a final concentration in 0.5 mM TDFHA, 25% (v/v) methanol.

AA	MH^+ (m/z)	RT (min)	Equation	R^2	Range
**Glycine**	76.04	0.92±0.04	$y=125.947x^2+107.416x+0.834$	0.996	1-1000 µM
**Alanine**	90.06	1.19±0.06	$y=5.449x^3+26.433x^2+460.063x$	0.989	0-6000 µM
**Serine**	106.05	0.83±0.04	$y=1901.870x^2-349.818x+45.721$	1	25-1000 µM
**Proline**	116.07	1.25±0.08	$y=3.518x-1.435$	1	1-25 µM
**Valine**	118.09	6.20±0.80	$y=0.062x^2+6.760x-1.757$	1	1-200 µM
**Threonine**	120.07	1.00±0.04	$y=1.233x^2+11.335x+4.254$	0.998	10-100 µM
**Isoleucine**	132.10	12.56±0.50	$y=0.040x^2-4.395x-1.190$	1	1-100 µM
**Leucine**	132.10	13.62±0.50	$y=0.055x^2+4.043x-1.454$	1	1-100 µM
**Asparagine**	133.06	0.80±0.04	$y=-0.062x^3+4.649x^2-2.928x+6.740$	1	1-3000 µM
**Aspartic acid**	134.05	0.68±0.04	$y=-1.068x^2+23.148x+0.478$	0.999	10-100 µM
**Glutamine**	147.08	0.89±0.04	$y=0.003x^3+0.455x^2+9.584x$	1	0-5000 µM
**Lysine**	147.11	16.15±0.30	$y=-1.004x^3+10.212x^2+11.380x$	0.999	0-200 µM
**Glutamic acid**	148.06	0.97±0.04	$y=0.537x^3-5.562x^2+27.782x-9.991$	1	10-1000 µM
**Methionine**	150.06	7.25±0.90	$y=6.716x$	0.999	0-100 µM
**Histidine**	156.08	16.05±0.16	$y=12.545x-3.007$	1	1-100 µM
**Phenylalanine**	166.09	15.70±0.18	$y=0.146x^2+2.900x-0.548$	1	1-100 µM
**Arginine**	175.12	16.20±0.14	$y=0.644x^2+5.015x$	1	0-200 µM
**Tyrosine**	182.08	9.53±0.40	$y=-0.526x^2+14.607x-0.512$	1	1-100 µM
**Tryptophan**	205.10	16.03±0.14	$y=58.193x^3-107.263x^2+67.771x-4.640$	0.999	1-25 µM
**Cysteine (2)**	241.03	1.13±0.08	$y=537.571x+0.574$	0.999	1-10 µM

Evaluation of soluble protein content

Additional Figure A4. Evaluation of soluble protein content in maize roots. Maize plants were grown for 10 days without N sources and then exposed for the last 30 h to absence of N (c), to 10 mM NO_3^- (n), to 10 mM NH_4^+ (a) or to 5 mM NO_3^-+5 mM NH_4^+ (na). Data are expressed as average values ±SE (n=3). The upper letters indicate differences among the four treatments according to Student's t-test (p≤0.01).

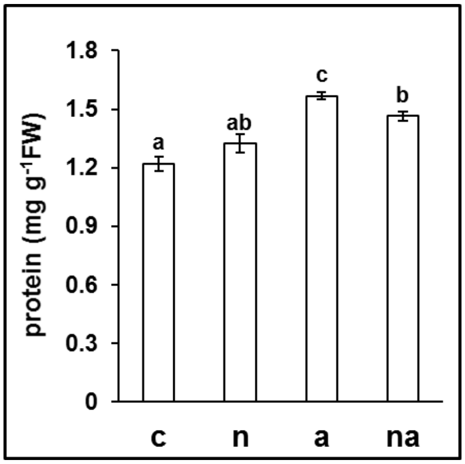

List of abbreviations: 1D: mono-dimensional, 2D: two-dimensional, (a): plants exposed to 10 mM NH_4^+, ALA: alanine, ARG: arginine, ASN: asparagine, ASP: aspartic acid, (c): control plants exposed to N absence, CYS: cysteine, GLN: glutamine, GLU: glutamic acid, GLY: glycine, GS: glutamine synthetase, HIS: histidine, ILE: isoleucine, LC-ESI-MS: Liquid Chromatography-ElectroSpray Ionization-Mass Spectrometry, LC-nESI-MS/MS: Liquid Chromatography-nanoElectroSpray Ionization-Tandem Mass Spectrometry, LEU: leucine, LYS: lysine, (n): plants exposed to 10 mM NO_3^-, N: nitrogen, (na): plants exposed to 5 mM NO_3^-+5 mM NH_4^+, NH_4^+: ammonium, NO_3^-: nitrate, PHE: phenylalanine, PRO: proline, MET: methionine, SER: serine, TDFHA: tridecafluoroheptanoic acid, THR: threonine, TRP: tryptophan, TYR: tyrosine, VAL: valine.
